# Heterozygous mutation of eEF1A1b resulted in spermatogenesis arrest and infertility in male tilapia, *Oreochromis niloticus*

**DOI:** 10.1038/srep43733

**Published:** 2017-03-07

**Authors:** Jinlin Chen, Dongneng Jiang, Dejie Tan, Zheng Fan, Yingying Wei, Minghui Li, Deshou Wang

**Affiliations:** 1 Key Laboratory of Freshwater Fish Reproduction and Development (Ministry of Education), Key Laboratory of Aquatic Science of Chongqing, School of Life Sciences, Southwest University, Chongqing, 400715, China

## Abstract

Eukaryotic elongation factor 1 alpha (eEF1A) is an essential component of the translational apparatus. In the present study, *eEF1A1b* was isolated from the Nile tilapia. Real-time PCR and Western blot revealed that eEF1A1b was expressed highly in the testis from 90 dah (days after hatching) onwards. *In situ* hybridization and immunohistochemistry analyses showed that eEF1A1b was highly expressed in the spermatogonia of the testis. CRISPR/Cas9 mediated mutation of *eEF1A1b* resulted in spermatogenesis arrest and infertility in the F_0_ XY fish. Consistently, heterozygous mutation of *eEF1A1b (eEF1A1b*^+/−^) resulted in an absence of spermatocytes at 90 dah, very few spermatocytes, spermatids and spermatozoa at 180 dah, and decreased Cyp11b2 and serum 11-ketotestosterone level at both stages. Further examination of the fertilization capacity of the sperm indicated that the *eEF1A1b*^+/−^ XY fish were infertile due to abnormal spermiogenesis. Transcriptomic analyses of the *eEF1A1b*^+/−^ testis from 180 dah XY fish revealed that key elements involved in spermatogenesis, steroidogenesis and sperm motility were significantly down-regulated compared with the control XY. Transgenic overexpression of *eEF1A1b* rescued the spermatogenesis arrest phenotype of the *eEF1A1b*^+/−^ testis. Taken together, our data suggested that *eEF1A1b* is crucial for spermatogenesis and male fertility in the Nile tilapia.

Eukaryotic translation elongation factor 1A (eEF1A) is one of the most abundant protein synthesis factors in eukaryotic cells. It is responsible for delivering aminoacylated tRNAs to the A site of the ribosome in a GTP-dependent reaction^1^. Eukaryotes possess a variable number of *eEF1A* genes with various expression patterns. Yeast (*Saccharomyces cerevisiae*) contains two *eEF1A* genes that are expressed almost equally in exponentially growing cells^2^. Fruit flies (*Drosophila melanogaster*) have two *eEF1A* genes, one of which is expressed only in certain life history stages^3^,^4^. In teleosts, only one *eEF1A* gene has been reported in zebrafish (*Danio rerio*), sea bream (*Sparus aurata*), Nile tilapia (*Oreochromis niloticus*) and medaka (*Oryzias latipes*)^5^,^6^,^7^,^8^. Recently, five *eEF1A* genes, referred to as *eEF1A1-4* and *42Sp50*, have been isolated and their relative expressions are different in ten tissues in flatfish (*Solea senegalensis*)^9^. In the African clawed frog (*Xenopus laevis*), three *eEF1A* genes are developmentally specific: the somatic form (*eEF-1S*) is present in embryos and most adult tissues, but is not detected in the germ cells; the oocyte form (*eEF-1O*), is present in germ cells and some adult tissues, but is not detected in the embryos; the *42Sp50* form, is detected only in oocytes^10^,^11^,^12^. In mammals, two *eEF1A* genes, named as *eEF1A1* and *eEF1A2*, have been identified, *eEF1A1*, which is almost ubiquitously expressed, and *eEF1A2*, whose expression is restricted to some cell types in a few tissues^13^,^14^,^15^,^16^. Despite this, homozygous mutation of mammalian *eEF1A2* is lethal during postnatal stages, possibly due to its critical function in translation elongation^17^,^18^,^19^.

Although eEF1A is reported to be ubiquitously expressed in all tissues examined, its role in gonads remains to be elucidated. Gametogenesis is a complex process, during which numerous proteins are synthesized in the testis and ovary. Mutation of the elongation factors for proteins synthesis, may result in infertility in vertebrates, as was demonstrated for eEF4^20^. Besides its canonical function in translation elongation, eEF1A is also involved in other cellular processes, such as severing microtubule^21^, bundling F actin^22^, controlling cell apoptosis^23^ and regulating protein degradation^24^,^25^. To date, only one report showed that *eEF1A* genes might be involved in the gametogenesis via non-canonical function. Oral administration of the eEF1A1 inhibitor gamendazole resulted in infertility in male mice, probably via disruption of actin-filament bundles related to the Sertoli cell-spermatids ectoplasmic specialization junctions^26^. However, there have been no other reports on the role and mechanism of other eEFs, including eEF1A, in gametogenesis.

In addition to somatic cells, eEF1A is also expressed in germ cells. Proteins expressed in germ cells are directly related to gametogenesis. Disruption of genes expressed in germ cells often blocks gametogenesis^27^,^28^,^29^,^30^. Therefore, eEF1A might be directly involved in gametogenesis via protein synthesis in germ cells. Different *eEF1A* genes are reported to be expressed in the testis and ovary of *Xenopus*^11^,^12^. Our gonads transcriptomic data from gonads demonstrated that *eEF1A1b* and *42Sp50* are XY-enhanced and XX-specific genes in tilapia, respectively^31^. Taken together, we speculate that different elongation factors might be used for protein synthesis during spermatogenesis and oogenesis in lower vertebrates. Species with different *eEF1A* genes expressed in the testis and ovary would be a good model to study the diverse function of the *eEF1A* genes in spermatogenesis and oogenesis.

Nile Tilapia (*Oreochromis niloticus*), is a good model for the study of gene expression and function in fish due to the availability of monosex fish^32^, a short spawning cycle, many gonadal transcriptomes^31^ and published genome sequences^33^. In the present study, expression of *eEF1A1b* was found to been enriched in XY males during tilapia gonadal development. Ontogenetic studies were conducted to determine its expression profile by real-time PCR and Western blot. The cellular localization of eEF1A1b was documented by *in situ* hybridization (ISH) and immunohistochemistry (IHC). We also mutated e*EF1A1b* and analyzed the gonadal phenotype, gene expression, serum 11-ketotestosterone (11-KT) level and fertility of the eEF1A1b deficiency and e*EF1A1b*^+/−^ fish.

## Results

### Phylogenetic and synteny analyses

As shown in the phylogenetic tree (supplemental Fig. S1), the phylogeny of eEF1A1 was split into two main clades. One clade included the eEF1A1 gene of tetrapods; and the other included the two distinct copies of eEF1A1 from teleosts that we named *eEF1A1a* and *eEF1A1b.*

Synteny analyses revealed the organization of the genomic region surrounding eEF1A1 genes in tetrapods and teleosts. *eEF1A1a* and its upstream gene *slc17a5*, its downstream gene *ddx43* and *kcnq5a* showed conserved synteny both in teleosts and tetrapods. *eEF1A1b* and its upstream gene *dtd1* showed conserved synteny only in teleosts, and its downstream gene *kcnq5b* and *rims1* showed conserved synteny only in tetrapods. Except *kcnq5*, no other conserved synteny of gene loci surrounding *eEF1A1a* and *eEF1A1b* were found (supplemental Fig. S2).

### Tissue distribution

Analysis by IHC showed that eEF1A1b was highly expressed in the brain, heart, liver, intestine, kidney and testis, weakly expressed in the ovary, but was undetectable in gill, spleen, head kidney and muscle (Supplemental Fig. S3).

### Expression profile of eEF1A1b in developing gonads

**Figure 1:**
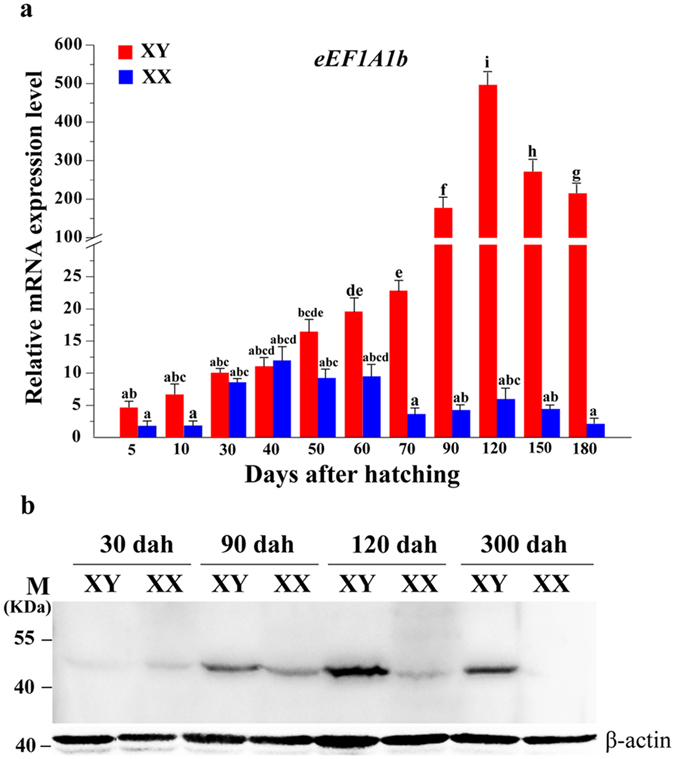
Expression patterns of eEF1A1b in developing gonads. (**a)** Ontogenetic expression of *eEF1A1b* in tilapia gonads analyzed by real-time PCR. Data are expressed as mean ± SD of three different gonadal pools at each developmental stage. Different letters indicate statistical differences (P < 0.05) as determined by one-way ANOVA followed by post hoc test. (**b**) Expression of eEF1A1b in gonads of XX and XY fish at 30, 90, 120 and 300 dah by Western blot. Lanes 1, 3, 5 and 7, proteins extracted from testes; lanes 2, 4, 6 and 8, proteins extracted from ovaries. eEF1A1b was detected in both testis and ovary with significant higher expression in testis. 0.2 micrograms of protein were added per lane.

Real-time PCR demonstrated that expression of *eEF1A1b* was low from 10 to 90 dah, increased sharply from 90 dah in testis and slightly decreased from 150 to 180 dah; while no significant changes were found during all stages in ovary (Fig. 1a).

Western blot analyses revealed that eEF1A1b was expressed in both testis and ovary, with significantly higher expression in the testis than in the ovary from 90 dah, peaking at approximately 120 dah, and decreased to a much lower level at 300 dah (Fig. 1b).

### Cellular localization of eEF1A1b in gonads

**Figure 2:**
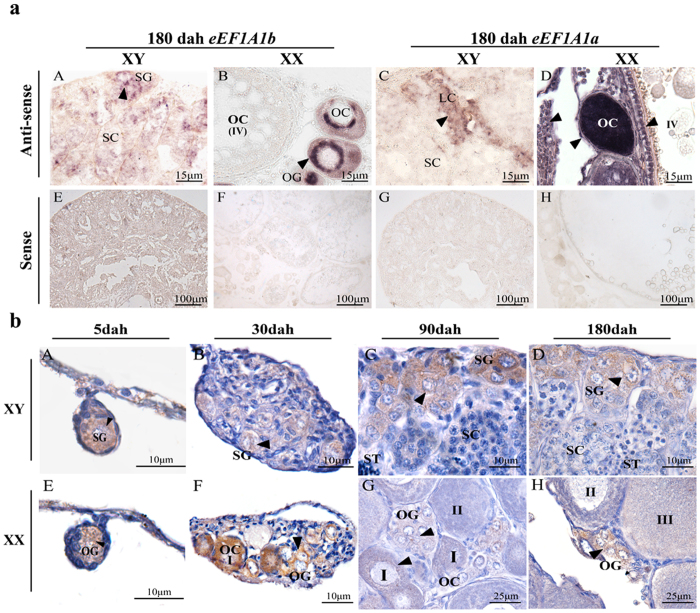
Cellular localization of eEF1A1b in tilapia testis and ovary at different developmental stages by ISH and IHC. (**a**) Cell type expressing *eEF1A1a* and *eEF1A1b* in tilapia gonads by ISH. *eEF1A1b* was detected in spermatogonia of the testis, while in the oogonia and phase I oocytes of the ovary at (A and B). *eEF1A1a* was detected in somatic cell of the testis, while in the oocytes and somatic cells of the ovary (C and D). (**b**) Cell type expressing eEF1A1b in tilapia gonads by IHC. Consistent with *in situ* hybridization results, eEF1A1b was detected in the spermatogonia of the testis from 5 to 180 dah (A–D), while in the oogonia of ovary at 5 dah, later in the oogonia and phase I oocytes of the ovary from 30 to 180 dah (E-H). SG, spermatogonia; SC, spermatocytes; ST, spermatids; OG, oogonia; OC, oocytes; I-IV, phase I to phase IV oocytes; Arrowheads indicate the positive signal.

ISH analysis demonstrated that *eEF1A1b* was expressed in the spermatogonia of the testis, and in the oogonia and phase I oocytes of the ovary with *eEF1A1b* antisense probe (Fig. 2a, A and B). *eEF1A1a*, the paralogous gene of *eEF1A1b*, was expressed in the somatic cells of the testis, and in the oocytes and somatic cell of the ovary (Fig. 2a, C and D). In contrast, signal was detected in neither testis nor ovary with *eEF1A1a* and *eEF1A1b* sense probe (Fig. 2a, E- H).

Consistently, IHC analysis revealed that eEF1A1b was expressed exclusively in the spermatogonia of the testis from 5, 30, 90 and 180 dah (Fig. 2b, A-D), and was expressed in the oogonia of the ovary at 5 dah, later in the oogonia and phase I oocytes of the ovary at 30 to 180 dah (Fig. 2b, E-H).

### Disruption of eEF1A1b by CRISPR/Cas9 and production of F_1_ generation

**Figure 3:**
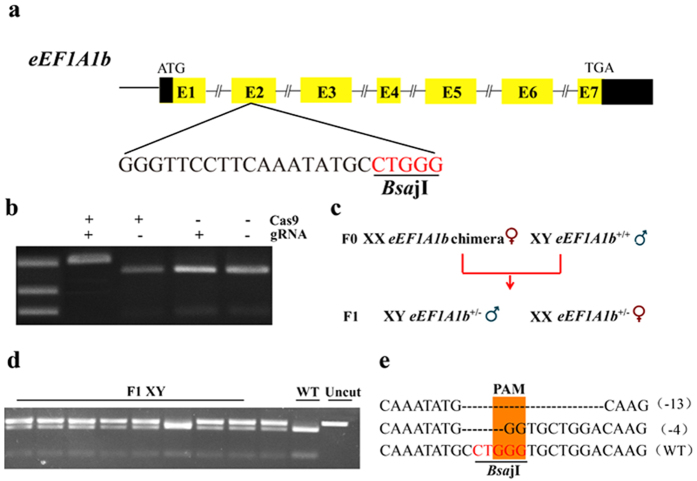
Design of CRISPR/Cas9 targeting *eEF1A1b* and establishment of the *eEF1A1b* mutation line. (**a,b)** Disruption of tilapia *eEF1A1b* by CRISPR/Cas9. Gene structure of *eEF1A1b* showing the target site and the *Bsa*jI restriction site. 300 ng/μl of Cas9 mRNA and 150 ng/μl of gRNA were co-injected into one-cell stage embryos. At 72 hours after injection, 20 embryos were randomly selected and pooled to extract their genomic DNA for PCR amplification. The indels were confirmed by two assays, restriction enzyme digestion and Sanger sequencing. The Cas9 mRNA and gRNA were added as indicated. Clear undigested band was detected in embryos injected with both Cas9 mRNA and gRNA compared with the control. (**c)** Schematic diagram showing the breeding plan of F_0_ to F_1_ fish. (**d)** *eEF1A1b*^+/−^ F_1_ generation detected by restriction enzyme digestion. (**e)** Sanger sequencing results from the uncleaved bands were listed. Deletions are marked by dashes, and the PAM is marked in light orange. Numbers to the right of the sequences indicate the loss or gain of bases for each allele, with the number of bases inserted (+) or deleted (−) indicated in parentheses. WT, wild type.

A guide RNA site containing *Bsa*jI adjacent to proto-spacer adjacent motif in the second exon of *eEF1A1b* was selected for mutation analysis (Fig. 3a). Genomic DNA from 20 pooled injected embryos and from fin of mutants was used as template for PCR amplification and mutation assays. Complete digestion produced fragments of 425 and 102 bp in the control groups whereas an intact DNA fragment was observed in embryos injected with both Cas9 mRNA and target guide RNA (Fig. 3b). The mutation rate in the pooled embryos was approximately 97%. In-frame and frame-shift deletions induced at the target site were confirmed by Sanger sequencing. We screened 40 individual microinjected fish by restriction enzyme digestion at 90 dah, and found 38 to be mutant for *eEF1A1b*. All mutant fish had mutation efficiency over 90% (Supplemental Tab. S3). Twenty mutant fish were further confirmed by Sanger sequencing. In total, we analyzed 200 sequences and identified 14 mutation types, including 11 types of deletion mutation and 3 types of insertion mutation. The majority (67.5%) were frame-shift mutations, while 32.5% were in-frame mutations (Supplemental Fig. S4). F_1_ offspring were obtained by outcross *eEF1A1b* chimeric XX with XY control fish, and two mutation types (4 and 13 bp deletion) were identified in F_1_ offspring by Sanger sequencing (Fig. 3c,d and e).

### Effects of eEF1A1b deficiency on spermatogenesis and fertility in F_0_ fish

**Figure 4:**
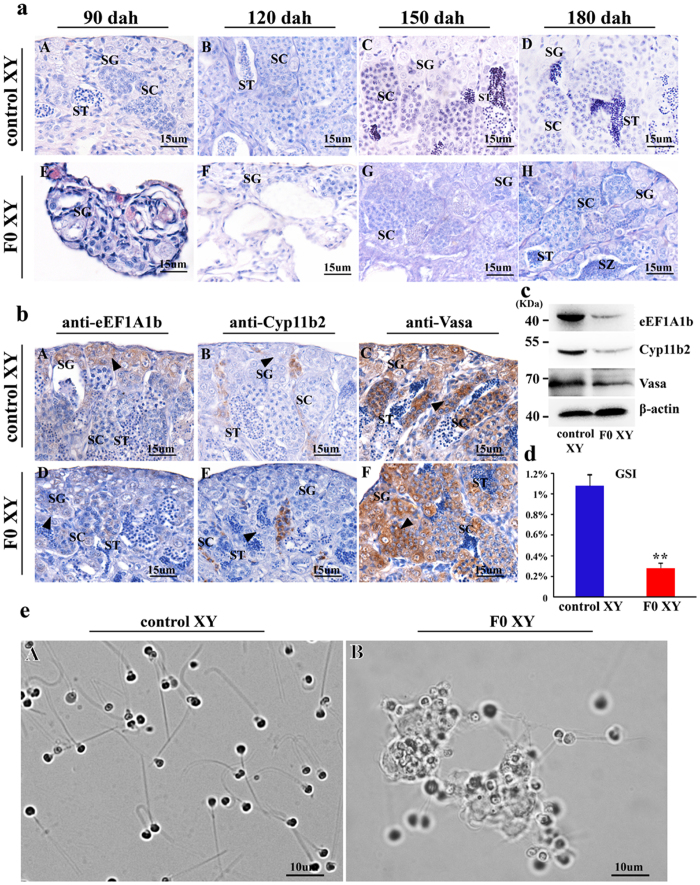
Effects of *eEF1A1b* deficiency on spermatogenesis and fertility in F_0_. (**a)** Histological observations of testis from F_0_ and control XY fish at 90, 120, 150 and 180 dah. *eEF1A1b* deficiency resulted in spermatogenesis arrest. All kinds of germ cells including spermatogonia, spermatocytes, spermatids and spermatozoa were present at the control testis at 90, 120, 150 and 180 dah (A-D). In contrast, in the F_0_ testis only spermatogonia were observed at 90 and 120 dah, spermatocytes appeared at 150 dah, and spermatids and spermatozoa appeared at 180 dah (E-H). (**b)** Expression of eEF1A1b, Cyp11b2 and Vasa in the F_0_ and control XY fish by IHC. By IHC, eEF1A1b was observed in the control testis (A) while almost undetectable in F_0_ testis (D). Reduced Cyp11b2 expression was observed in Leydig cells in the F_0_ testis (B) compared with the control testis (E). Vasa positive signals were detected in both the control and F_0_ testis (C and F). Arrowheads indicate the positive signal. SG, spermatogonia; SC, spermatocytes; ST, spermatids; SZ, spermatozoa. (**c**) Expression of eEF1A1b, Cyp11b2 and Vasa in the F_0_ and control XY fish by Western blot. (**d)** Gonadal somatic index (GSI) of the F_0_ and control XY fish. (**e**) Morphology and motility of sperm from 180 dah F_0_ and control XY fish. The sperm from the control fish were with normal flagella, and displayed vigorous flagella activity and progressive movement (A); while the sperm from F_0_ fish were characterized by short or absent flagella. The motility of short-flagella sperms was weak, and the flagella-less sperms were stuck together, showing no motility (B). F_0_, eEF1A1b deficiency.

Morphologically, all kinds of germ cells including spermatogonia, spermatocytes, spermatids and spermatozoa were presented in the control testis at 90, 120, 150 and 180 dah (Fig. 4a, A-D). In contrast, in the eEF1A1b deficient testis only spermatogonia were observed at 90 and 120 dah, spermatocytes appeared at 150 dah and spermatids and spermatozoa appeared at 180 dah (Fig. 4a, E-H). Additionally, fewer spermatozoa were observed in the efferent duct of the eEF1A1b deficient testis at 180 dah compared with the control.

By IHC, eEF1A1b was detected in the spermatogonia, and Cyp11b2 was observed in the Leydig cells in the control testis at 180 dah (Fig. 4b, A and B). In contrast, reduced expression of eEF1A1b and Cyp11b2 was observed in the eEF1A1b deficient testis (Fig. 4b, D and E). However, Vasa was detected in the spermatogonia and spermatocytes in the eEF1A1b deficient and control testis (Fig. 4b, C and F).

Western blot analyses revealed that the expression of eEF1A1b, Cyp11b2 and Vasa was significantly reduced in the eEF1A1b deficient testis compared with those of the control (Fig. 4c). Additionally, the size of the eEF1A1b deficient testis was significantly smaller than that of the control testis as demonstrated by gonadal somatic index (GSI, gonad weight/body weight × 100%) (Fig. 4d).

Fertility test showed that the eEF1A1b deficient XY fish were infertile (supplemental Tab. S4). When examined under microscope, the sperm of the eEF1A1b deficient fish were abnormal. The sperm from the control fish were normal with a flagella length of 16.2 ± 2.4 μm, and displayed strong motility. In contrast, the sperm from eEF1A1b deficient fish were characterized by short or absent flagella. The short-flagella sperms with a flagella length of 7.55 ± 3.16 μm, displayed weak motility, and the flagella-less sperms were stuck together, showing no motility (Fig. 4e; supplemental Fig. S5).

### Effects of eEF1A1b deficiency on ovarian development in F_0_ fish

In contrast to the significant phenotypes of eEF1A1b deficiency in the testis, the ovary of most mutant fish showed normal development at 120 and 180 dah. Histological analysis demonstrated that folliculogenesis was normal in the ovary of the eEF1A1b deficient XX fish (Supplemental Fig. S6).

### Effects of eEF1A1b heterozygous mutation on spermatogenesis and fertility in F_1_ fish

**Figure 5:**
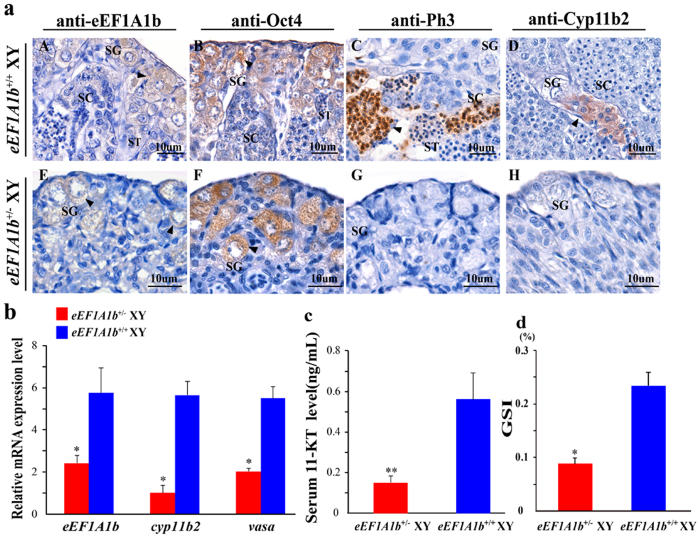
Effects of the heterozygous mutation of *eEF1A1b* on spermatogenesis and fertility. (**a**) IHC analyses of eEF1A1b, Oct 4 (spermatogonia maker), Ph3 (spermatocyte maker) and Cyp11b2 expression in the testis of *eEF1A1b*^+/−^ and *eEF1A1b*^+/+^ XY fish at 90 dah. eEF1A1b and Oct 4, Ph3, Cyp11b2 were observed in the spermatogonia, spermatocytes and Leydig cells, respectively, of the *eEF1A1b*^+/+^ testis. In contrast, Oct 4 and reduced expression of eEF1A1b were observed in the spermatogonia, while no Ph3 and Cyp11b2 expression was detected in the *eEF1A1b*^+/−^ testis. Arrowheads indicate the positive signal. SG, spermatogonia; SC, spermatocytes; ST, spermatids. (**b)** Expression of *eEF1A1b, cyp11b2* and *vasa* in the *eEF1A1b*^+/−^ and *eEF1A1b*^+/+^ XY fish at 90 dah by real-time PCR. (**c)** Serum 11-KT levels of the *eEF1A1b*^+/−^ and *eEF1A1b*^+/+^ XY fish. (**d)** GSI of *eEF1A1b*^+/−^ and *eEF1A1b*^+/+^ XY fish at 90 dah. Results were expressed as mean ± SD. Different letters indicate statistical differences at P < 0.05 as determined by one-way ANOVA followed by post hoc test.

The heterozygous mutation of *eEF1A1b (eEF1A1b*^+/−^) XY fish with a 4 bp deletion were employed for phenotype analyses. Consistent with the phenotype of F_0_ generation, *eEF1A1b*^+/−^ XY fish also displayed spermatogenesis arrest. Histologically, spermatogonia, spermatocytes and spermatids, were present at *eEF1A1b*^+/+^ testis, whereas only spermatogonia were observed in *eEF1A1b*^+/−^ XY testis at 90 dah (Fig. 5a). By IHC, eEF1A1b and Oct 4 (spermatogonia maker), Phospho-histone h3 (Ph3, spermatocytes maker), Cyp11b2 were observed in the spermatogonia, spermatocytes and Leydig cells, respectively, of the *eEF1A1b*^+/+^ testis. In contrast, Oct 4 and reduced expression of eEF1A1b were observed in the spermatogonia, while no Ph3 and Cyp11b2 expression was detected in the *eEF1A1b*^+/−^ testis. These results indicated that there were only spermatogonia, but no spermatocytes in the *eEF1A1b*^+/−^ testis (Fig. 5a). In addition, the *eEF1A1b, cyp11b2* and *vasa* mRNA levels were found to be significantly down-regulated in the *eEF1A1b*^+/−^ testis by real-time PCR (Fig. 5b). Further, significantly lower serum 11-KT level was detected in the *eEF1A1b*^+/−^ XY fish compared with that of the *eEF1A1b*^+/+^ XY fish at 90 dah (Fig. 5c). Furthermore, the size of the *eEF1A1b*^+/−^ testis was significantly smaller than that of the *eEF1A1b*^+/+^ testis as reflected by the GSI.

**Figure 6:**
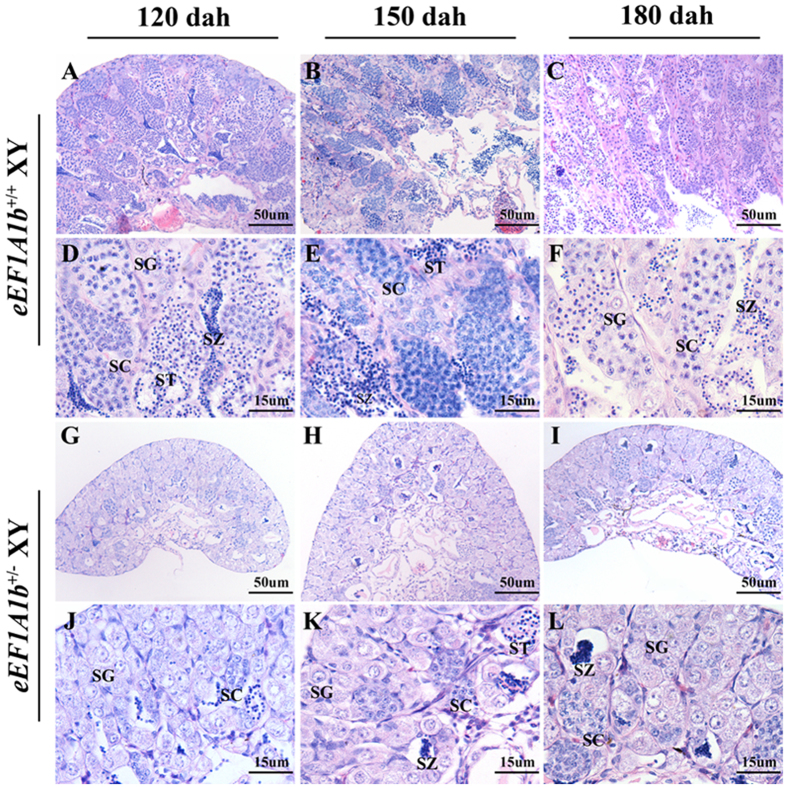
Histological observations of testis from *eEF1A1b*^+^/^−^ XY fish at 120, 150 and 180 dah. Abundant spermatocytes, spermatids and spermatozoa were observed in the testis of *eEF1A1b*^+/+^ XY fish at 120, 150 and 180 dah (A-F), while just very few spermatocytes were observed at 120 dah, and a markedly reduced number of spermatocytes, spermatids and spermatozoa were observed at 150 and 180 dah in the testis of *eEF1A1b*^+/−^ XY fish (G-L). D-F and J-L, Higher magnification of the A-C and G-I, respectively. SG, spermatogonia; SC, spermatocytes; ST, spermatids; SZ, spermatozoa.

By histology, abundant spermatocytes, spermatids and spermatozoa were observed in the *eEF1A1b*^+/+^ testis at 120, 150 and 180 dah (Fig. 6, A-F). In contrast, in the testis of *eEF1A1b*^+/−^ XY fish, just a few spermatocytes were observed at 120 dah, and a markedly reduced number of spermatocytes, spermatids and spermatozoa were observed at 150 and 180 dah (Fig. 6, G-L).

**Figure 7:**
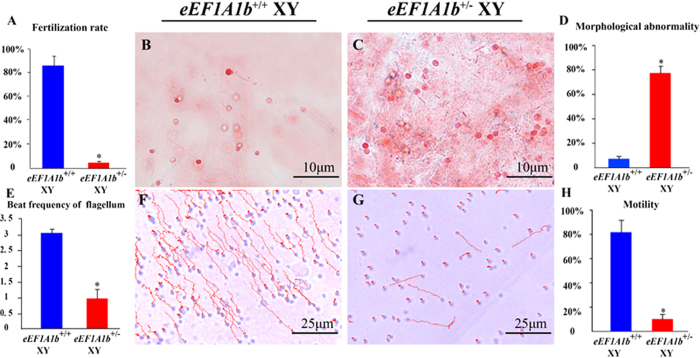
Sperm analyses of *eEF1A1b*^+^/^−^ XY fish at 180 dah. The fertilization rate of *eEF1A1b*^+/−^ XY fish was significantly lower than that of *eEF1A1b*^+/+^ XY fish (**A**). Sperm of *eEF1A1b*^+/+^ XY fish were with normal flagella, while most sperm of *eEF1A1b*^+/−^ XY fish were flagella-less, and the ratio of sperm with morphological abnormalities to the total sperm was higher in *eEF1A1b*^+/−^ XY fish than in *eEF1A1b*^+/+^ XY fish (**B-D**). The beat frequency of sperm flagella was higher in *eEF1A1b*^+/+^ XY fish than in *eEF1A1b*^+/−^ XY fish (**E**). And the ratio of forward motility sperm was higher in *eEF1A1b*^+/+^ XY fish than in *eEF1A1b*^+/−^ XY fish (**F-H**). The red line indicates the trajectories of sperm. Results were expressed as mean ± SD. Different letters indicate statistical differences at P < 0.05 as determined by one-way ANOVA followed by post hoc test.

Fertility test showed that similar to the chimeric XY fish, *eEF1A1b*^+/−^ XY fish were infertile (Fig. 7A). Morphological and biochemical analyses of sperm were performed to analyze the reason for male infertility in *eEF1A1b*^+/−^ XY fish. The sperm of *eEF1A1b*^+/+^ XY fish were characterized by normal flagella, while the sperm of *eEF1A1b*^+/−^ XY fish were flagella-less. Therefore, the ratio of sperm with morphological abnormalities to the total sperm was significantly higher in *eEF1A1b*^+/−^ XY fish than that in the *eEF1A1b*^+/+^ XY fish (Fig. 7, B-D). Consistently, the activity of sperm flagella was significantly lower in *eEF1A1b*^+/−^ XY fish than that in *eEF1A1b*^+/+^ XY fish (Fig. 7E). Consequently, the sperm forward motility was lost in the *eEF1A1b*^+/−^ XY fish (Fig. 7, F-H).

### Gonadal transcriptome analysis of eEF1A1b^+/−^ XY fish

**Figure 8:**
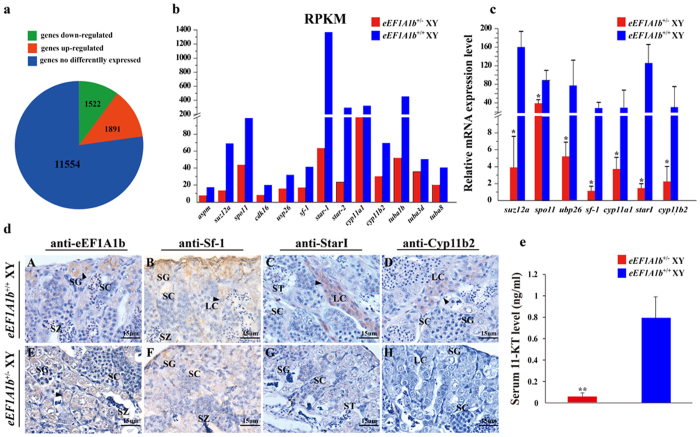
Transcriptomic analyses of gene expression profiles in *eEF1A1b*^+^/^−^ testis at 180 dah. (**a)** Testis expressed genes were divided into three parts: 1522 down-regulated genes, 1891 up-regulated genes, and 11554 non-differentially expressed genes. (**b)** Validation of genes with disrupted expression files from transcriptome data by real-time PCR. All examined genes displayed similar expression profiles to those from the transcriptome data. (**c)** Validation of genes with disrupted expression files from transcriptome data by IHC. Arrowheads indicate the positive signal. SG, spermatogonia; SC, spermatocytes; ST, spermatids; SZ, spermatozoa; LC, Leydig cells. (**d**) Serum 11-KT levels of the *eEF1A1b*^+/−^ and *eEF1A1b*^+/+^ XY fish.

Transcriptome sequencing of the testes from the *eEF1A1b*^+/−^ XY fish at 180 dah yielded a total of 40,039,839 reads. The *eEF1A1b*^+/−^ XY transcriptome data were analyzed using the transcriptome data from testis of 180 dah XY fish obtained earlier by our group^31^ as a control. The total gene counts of the *eEF1A1b*^+/−^ XY fish (22,284) were slightly more than those of the *eEF1A1b*^+/+^ XY fish (21,365). Compared with the control testis, 1,522 genes were down regulated, 1,891 genes were up regulated, 11,554 genes were not differentially expressed and 7,317 genes were at background expression level in the *eEF1A1b*^+/−^ XY testis (Fig. 8a).

Further analysis revealed that of the 1522 down-regulated genes, 416 genes were involved in spermatogenesis process (e.g. *aspm, cdk16, suz12a, usp26* and *spo11*), 86 genes were involved in steroidogenesis (e.g. *sf-1, cyp11a1, star* and *cyp11b2*) and 19 genes were involved in sperm motility (e.g. *tuba1b, tuba3d* and *tuba8*) (Fig. 8b). Real-time PCR analyses showed that mRNA levels of *suz12a, spo11, usp26, sf-1, cyp11a1, star1* and *cyp11b2* were significantly down-regulated in the *eEF1A1b*^+/−^ testis compared with the control (Fig. 8c).

By IHC, eEF1A1b were observed in the spermatogonia, and Sf-1, StarI and Cyp11b2 were observed in the Leydig cells of the *eEF1A1b*^+/+^ testis (Fig. 8d, A-D). In contrast, reduced eEF1A1b and Sf-1 expression and very sparse StarI and Cyp11b2 were detected in the *eEF1A1b*^+/−^ testis (Fig. 8d, E-H). Consequently, the serum 11-KT level of the eEF1A1b^+/−^ XY fish was significantly lower than that of *eEF1A1b*^+/+^ XY fish (Fig. 8e).

### Transgene mediated rescue of spermatogenesis in *eEF1A1b* ^+/−^ XY fish

**Figure 9:**
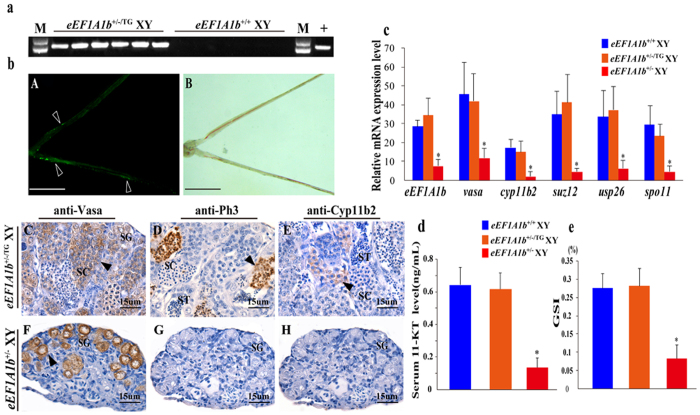
Transgene mediated rescue of spermatogenesis in *eEF1A1b*^+^/^−^ XY fish. (**a)** Confirmation of *eEF1A1b* transgene insertion by genomic PCR. Lane 1 and 14, DNA marker; Lane 2-7, XY fish carrying *eEF1A1b*-transgene; Lane 8-13, XY control fish; Lane 15, empty plasmid as template. (**b**) A, the GFP signals in the testis of *eEF1A1b*^+/−/TG^ XY fish in a dark field; Arrowheads indicate the positive signal. B, bright field; C-H, IHC analyses of Vasa, PH3 and Cyp11b2 expression in the testis of *eEF1A1b*^+/−/TG^ and *eEF1A1b*^+/−^ XY fish; SG, spermatogonia; SC, spermatocytes; ST, spermatids. (**c**) Expression of *eEF1A1b, cyp11b2, vasa, suz12, usp26* and *spo11* in the *eEF1A1b*^+/−/TG^, *eEF1A1b*^+/−^ and *eEF1A1b*^+/+^ XY fish by real-time PCR. (**d**) Serum 11-KT levels of the *eEF1A1b*^+/−/TG^, *eEF1A1b*^+/−^ and *eEF1A1b*^+/+^ XY fish. (**e**) GSI of *eEF1A1b*^+/−/TG^, *eEF1A1b*^+/−^ and *eEF1A1b*^+/+^ XY fish. Results were expressed as mean ± SD. Different letters indicate statistical differences at P < 0.05 as determined by one-way ANOVA followed by post hoc test.

To rescue the spermatogenesis arrest phenotype of *eEF1A1b*^+/−^ XY fish, transgenic overexpression of *eEF1A1b* was conducted in the *eEF1A1b*^+/−^ XY fish using a CMV promoter-derived expression vector. A specific band of 720 bp was amplified from the genomic DNA of *eEF1A1b* transgenic (*eEF1A1b*^+/−/TG^) fish (Fig. 9a). GFP signals were detected in the *eEF1A1b*^+/−/TG^ testis when examined at 90 dah (Fig. 9b, A and B).

By IHC, Vasa and Ph3 were observed in the spermatogonia/spermatocytes and spermatocytes, and Cyp11b2 were detected in the Leydig cells of the *eEF1A1b*^+/−/TG^ testis (Fig. 9b, C-E). In contrast, reduced Vasa, and no Ph3 and Cyp11b2 were observed in the *eEF1A1b*^+/−^ testis (Fig. 9b, F-H).

By real-time PCR, the expression of *eEF1A1b, cyp11b2, vasa, suz12* and *usp26* in the *eEF1A1b*^+/−/TG^ testis showed no significant differences compared with the *eEF1A1b*^+/+^ testis, but significantly higher than that of the *eEF1A1b*^+/−^ testis (Fig. 9c). Consistently, the serum 11-KT level and GSI of the *eEF1A1b*^+/−^ XY fish showed no significant differences compared with the *eEF1A1b*^+/+^ testis, but significantly higher than those of the *eEF1A1b*^+/−^ testis (Fig. 9d and e)

## Discussion

### Evolutionary implication of eEF1A1b

In the present study, *eEF1A1a and eEF1A1b* genes were successfully identified from the Nile tilapia, zebrafish, medaka and fugu. Phylogenetic analyses of eEF1A1s from vertebrates demonstrated that *eEF1A1a* and *eEF1A1b* arose from the teleost genome duplication^34^. *eEF1A1a* has retained the ancestral functions, while *eEF1A1b* has evolved novel function/expression patterns. Moreover, conserved synteny of *eEF1A1a* with *kcnq5a* and *eEF1A1b* with *kcnq5b* was observed in teleosts. Taken together, these results suggested that *eEF1A1a* and *eEF1A1b* were derived from the teleost-specific genome duplication.

### The expression pattern and functional relevance of eEF1A1b in tilapia

Spatial-temporal gene expression patterns are important aspects of gene function analysis. Our previous data showed that *eEF1A1a* displayed no gender difference in expression, while *eEF1A1b* was a male-biased gene in tilapia gonad^31^. In the present study, both real-time PCR and Western blot analyses demonstrated that *eEF1A1b* showed sexually dimorphic expression from 70 dah, and was highly expressed in the testis from 90 dah onwards, correlated with the initiation of spermatogenesis and the appearance of spermatocytes. The expression of *eEF1A1b* reached its highest levels with the increase of the spermatogonia at 120 dah. Further, *eEF1A1b* was expressed exclusively in spermatogonia, while *eEF1A1a* was found to be mostly expressed in somatic cells of the testis. In the ovary, the expression of *eEF1A1b* was low and constant. *eEF1A1* is a housekeeping gene required for survival of all eukaryotic cells^35^,^36^, and therefore, at least one *eEF1A* gene must be expressed in the germ cells and somatic cells of the gonad in vertebrates. In mammals, *eEF1A1* is a sex unbiased gene, and is expressed in the germ cell and somatic cell of the gonad^13^,^14^,^15^,^16^. In *Xenopus, eEF-1S* and *eEF-1O* are expressed in somatic cells and germ cells respectively, and *42sp50* is detected only in oocytes^10^,^11^. In tilapia, *eEF1A1a* was expressed in the somatic cells of the testis and ovary, while *eEF1A1b* and *42Sp50* were expressed in the spermatogonia and oogonia/oocytes, respectively. Taken together, different elongation factor isoforms are used for male and female germ cells, as well as germ cells and somatic cells in lower vertebrates, while only one (*eEF1A1*) is used in both male and female germ cells and somatic cells of gonad in mammals.

In the present study, eEF1A1b deficiency resulted in spermatogenesis arrest and consequent infertility in F_0_ XY fish. This was reflected by defective of meiosis, reduced GSI and abnormal spermiogenesis. Heterozygous mutants of *eEF1A1b* displayed a phenotype similar to that of the F_0_ XY fish. The *eEF1A1b*^+/−^ testis showed no and very few spermatocytes at 90 and 120 dah, respectively. Spermatocytes, spermatids and spermatozoa were remarkably reduced at 150 and 180 dah. Further, most sperm from the *eEF1A1b*^+/−^ XY fish were flagella-less, and therefore, incapable of fertilization. Anatomical examination of *eEF1A1b*^+/−^ XY fish showed mostly normal organs and tissues, including the heart and brain, the organ with high eEF1A1b abundance (supplemental Fig. S7). Thus, our results indicated the impairment of testis development, and failure of fertilization in mutant fishes were probably not the result of defects in these tissues, but a specific effect of the *eEF1A1b* deficiency in testis. Both the eEF1A1b deficient and *eEF1A1b*^+/−^ XY fish were infertile, clearly indicating that *eEF1A1b* is critical for spermatogenesis and fertility in male tilapia.

Of all eEF1A isoforms, only *eEF1A1b* was expressed in the spermatogonia in tilapia. Consequently, mutation of *eEF1A1b* caused spermatogenesis arrest and infertility. It is well documented that eEF1A is indispensable for protein synthesis. Recently, a report showed that genetic ablation of eEF4, which is a key quality-control factor in translation, causes testis-specific dysfunction in oxidative phosphorylation, leading to male infertility in mice^20^. Besides elongation factors, disruption of other genes expressed in the spermatogonia also blocks spermatogenesis^27^,^29^,^37^. Consistently, in the present study, the mRNA level of *suz12a, aspm*, and *usp26*, which were expressed in spermatogonia and critical for spermatogenesis^28^,^37^,^38^,^39^, were significantly down regulated in the *eEF1A1b*^+/−^ testis. It is reasonable to believe that they were also down regulated in protein levels although we did not check the protein levels of these genes due to lack of specific antibodies. On the other hand, eEF1A1 might also be involved in spermatogenesis via other cellular processes^26^. However, the non-canonical function of *eEF1A1b* in spermatogenesis of tilapia remains to be elucidated.

In addition, suppression of steroidogenesis was observed in *eEF1A1b* mutant XY fish, as reflected by the decrease of Leydig cells, down-regulation of the expression of Sf-1 and the steroidogenic enzyme StarI, StarII, Cyp11a1, Cyp11b2 and 11β-HSD and a decrease in serum 11-KT level. It is well known that 11-KT is essential for spermatogenesis in fish^40^, and therefore, spermatogenesis arrest and infertility in eEF1A1b deficient and *eEF1A1b*^+/−^ XY fish might be the resulted of low levels of 11-KT. Nevertheless, it is an indirect effect of the *eEF1A1b* mutation as this gene is not expressed in the Leydig cells of tilapia. These results suggested that spermatogonia-derived signals might affect the Leydig cells directly or indirectly. It is widely accepted that, during the development of testis, male germ cells are influenced by signals from the surrounding somatic cells, but not vice versa^41^. But this conventional perspective was challenged recently. For example, mutation dominant-white spotting locus, which is expressed in spermatogonia of testis^42^, resulted in absence of germ cells, decreased Leydig cell population and disordered expression profiles of Leydig cells key markers (*cyp11a1, hsd3β* and *insl3*) in mice^43^. Moreover, overexpression of *figla* resulted in up-regulation of 11-KT in male tilapia^44^. Taken together, we speculate that the Leydig cells in the testis might be influenced by signals from the male germ cells. However, the mechanisms involved remain to be elucidated.

The phenotype of *eEF1A1b*^+/−^ testis was rescued by *eEF1A1b* overexpression as reflected by the normal testis size and appearance of all types of male germ cells, including spermatocytes and spermatids, which were absent in the *eEF1A1b*^+/−^ testis. Moreover, *eEF1A1b* transgene also rescued the expression of those genes involved in the spermatogenesis process and steroidogenesis altered in the *eEF1A1b*^+/−^ testis. These results validated that the spermatogenesis arrest phenotype is exclusively caused by *eEF1A1b* haploinsufficiency rather than off-target effects.

Previous reports showed that asthenozoospermia is a common cause of human male infertility, diagnosed by shortened sperm flagella, reduced sperm concentration and motility^45^ similar to the infertile phenotype of *eEF1A1b* mutant XY fish. The exact cause of asthenozoospermia is unclear so far, and mutation of eEF1A1 might be lethal in mammals. Mutation of *eEF1A1b* in tilapia could be used as a model for investigating the possible involvement of eEF1A1 in asthenozoospermia.

In summary, phylogenetic and syntenic analyses revealed that eEF1A1b is a duplicated copy of eEF1A1. In the testis it is exclusively expressed in the spermatogonia. Loss of function and transgenic overexpression studies indicated that the spermatogenesis arrest and infertility phenotypes observed in the *eEF1A1b*^+/−^ XY fish was indeed caused by *eEF1A1b* haploinsufficiency. Taken together, our results suggested that *eEF1A1b* is essential for spermatogenesis and male fertility in the Nile tilapia. In addition, mutation of eEF1A1b in tilapia may provide an excellent model for studying human asthenozoospermia.

## Materials and Methods

### Animals

Nile tilapia, *Oreochromis niloticus*, was kept in recirculating aerated freshwater tanks at 26 °C under a natural photoperiod. All-XX progenies were obtained by crossing a pseudomale (XX male, producing sperm after hormonal sex reversal) with a normal female (XX). All-XY progenies were obtained by crossing a supermale (YY) with a normal female. Animal experiments were conducted in accordance with the regulations of the Guide for Care and Use of Laboratory Animals and were approved by the Committee of Laboratory Animal Experimentation at Southwest University.

### Phylogenetic and synteny analysis of eEF1A1b

A phylogenetic tree of eEF1A1s was constructed using tilapia 42Sp50 as the outgroup. Sequences were obtained from the NCBI (http://blast.ncbi.nlm.nih.gov/) and Ensembl (http://www.ensembl.org/index.html) databases. The accession numbers of the sequences used are listed in the Supplemental Tab.S1. Full-length amino acid sequences were aligned using MEGA5.0^46^. The credibility of the branching was tested using bootstrap resampling with 1000 pseudo-replicates.

For syntenic analysis, the location of *eEF1A1* (including *eEF1A1a* and *eEF1A1b*) and their adjacent genes were determined for human, rat, chicken, green anole, clawed frog, Nile tilapia, medaka, fugu and zebrafish using the Ensembl genome browser (http://www.ensembl.org/index.html).

### Tissue distribution of eEF1A1b by IHC

The various tissues obtained from XX and XY tilapia at 180 dah tilapia were fixed in Bouin’s solution for 12 hours at room temperature, dehydrated and embedded in paraffin. All tissue blocks were sectioned at 5  μm and used for IHC analysis, which was performed as described previously^27^. The antibody against eEF1A1b was diluted 1:1000 for use.

### Production and characterization of eEF1A1b antibody

The peptide antigen (SGWNGDNMLEPSPNMT) of tilapia eEF1A1b was prepared and injected into rabbits. eEF1A1b antiserum (a-DTc) was collected and affinity purified. To confirm the specificity of the polyclonal antibody, total protein was extracted from XX and XY gonads of 30, 90, 120 and 300 dah tilapia. Western blots were carried out using the purified antibody at dilution of 1:500.

### Expression profile of eEF1A1b in tilapia gonad by real-time PCR and Western blot

For ontogenic expression analysis, three parallel gonadal samples were prepared from the fish at 5, 10, 30, 40, 50, 60, 70, 90, 120, 150 and 180 dah. For sampling points from 5 to 60 dah, fish were dissected and viscera were removed under a stereoscopic microscope. RNAlater reagent (Ambion, TX) was poured on the coelomic epithelium to stabilize the RNA in the gonads, and then the gonads were removed using fine forceps. Gonads were pooled in a tube with RNAlater reagent and stored at −80 °C before RNA extraction.

Total RNAs (2.0 μg) were extracted and reverse transcribed into cDNA using PrimeScript RT Master Mix Perfect Real Time Kit according to the manufacturer’s instructions (Takara, Japan). Real-time PCR was carried out on an ABI-7500 real-time PCR machine according to the protocol of SYBR^®^ Premix Ex Taq II. The relative abundance of *eEF1A1b* mRNA transcripts was evaluated using the formula: R = 2^−∆∆Ct^ 47. The geometric mean of the copy numbers of the three reference genes (*β-actin, gapdh* and *eEF1A1a)* was used to normalize the expression of *eEF1A1b*. Primer sequences used for real-time PCR are listed in Supplemental Tab. S2. Data were expressed as the mean ± S.D. Statistical analysis was performed using GraphPad Prism4 software (GraphPad Software, USA). Significant differences between groups were tested by one-way ANOVA with post-hoc test. P < 0.05 was considered to be statistically significant.

Total proteins extracted from testes and ovaries of 30, 90, 120 and 300 dah tilapia were separated using 12% SDS-PAGE under reducing condition. Western blot was carried out as reported previously^48^ using the purified antibodies at dilution of 1:500.

### Cellular location of eEF1A1b in gonads analysis by ISH and IHC

ISH was performed using tilapia gonads collected 180 dah from monosex (XX or XY) fish. Gonads were dissected and fixed in 4% paraformaldehyde in 0.1 M phosphate buffer (pH 7.4, 4% PFA) at 4 °C overnight. After fixation, gonads were embedded in paraffin. Cross-sections of 5 μm were cut with a sliding microtome and adhered to polylysine-treated slides with diethylpyrocarbonate-treated water. Slides were dried at 180 °C for 4 hours to remove the RNase contamination and stored at 4 °C until use. Probes for both sense and antisense digoxigenin-labeled RNA strands were transcribed *in vitro* from a linearized pGEM-Teasy-eEF1A1b cDNA clone using the RNA labeling kit (Roche, Germany). ISH was performed as described previously^49^.

For IHC analysis, the gonads of 5, 30, 90 and 180 dah monosex (XX and XY) fish were dissected, fixed in Bouin’s solution for 12 hours at room temperature, dehydrated and embedded in paraffin. All tissue blocks were sectioned at 5 μm for IHC analysis, which was performed as described previously^27^. The antibody against eEF1A1b was diluted 1:1000 for use.

### Disruption of *eEF1A1b* by CRISPR/Cas9

CRISPR/Cas9 was performed to knockout *eEF1A1b* in tilapia^27^. The gRNA target site was selected from *eEF1A1b* sequences corresponding to GGN18NGG on the sense or antisense strand of DNA (http://zifit.partners.org/ZiFiT/). Candidate target sequences were compared with the entire tilapia genome using the Basic Local Alignment Search Tool to avoid cleavage of off-target sites. One-cell stage embryos were divided into two batches, one for microinjection and the other for control. The gRNA and Cas9 mRNA were co-injected into one-cell stage embryos with the optimal concentration of 150 ng/μl and 300 ng/μl, respectively. Twenty injected embryos were collected 72 hours after injection. Genomic DNA was extracted from pooled injected embryos and control embryos, and used to access the mutations. DNA fragments spanning the *eEF1A1b* target site was amplified using gene specific primers (Supplemental Tab. 2). The indels were confirmed by restriction enzyme digestion with *Bsa*jI and Sanger sequencing. In addition, the percentage of uncleaved band was measured by quantifying the band intensity using Quantity One Software (Bio-Rad, USA)^50^. The indel (insertion and deletion) frequency was calculated by dividing uncleaved band intensity to the total band intensity from single digestion experiment.

The remaining micro-injected fish were reared until sampling for phenotypic assays. To screen the mutant fish, a piece of tail fin was clipped from each individual, and genomic DNA was extracted as described above. The target genomic locus was amplified using the primers eEF1A1b-cas-F/R. Mutations were assessed by restriction enzyme digestion and Sanger sequencing. The genetic sex of the mutants was determined using a sex-linked DNA marker (marker5) reported previous by our group^32^. The eEF1A1b-deficient fish were used for gonad histology, gene expression (real-time PCR, IHC, and Western blot), serum androgen (11-KT) analyses and fertility test at 90 and 180 dah.

### *eEF1A1b* deficiency on spermatogenesis and fertility

Gonads of eEF1A1b deficient fish were sampled at 90, 120, 150 and 180 dah. Samples were fixed in Bouin’s solution for 24 hours at room temperature, dehydrated and embedded in paraffin. Tissue blocks were sectioned at 5 μm and stained with hematoxylin and eosin (H.E) or used for IHC analysis. Vasa (germ cell marker) antibody was donated by Prof. Nagahama, the National Institute of Basic Biology, Okazaki, Japan. The specificity of the antibody has been analyzed previously^51^. eEF1A1b and Cyp11b2 (11β-hydroxylase, the key enzyme for androgen 11-KT synthesis) antibody were prepared by our laboratory. The specificity of Cyp11b2 antibody was checked previously^52^. Antibodies against eEF1A1b, Vasa and Cyp11b2 were diluted 1:1000, 1:1000 and 1:500 for use, respectively. IHC analyses were performed as described above. Photographs were taken under Olympus BX51 light microscope.

Western blots were performed to confirm the expression change of eEF1A1b, Cyp11b2 and Vasa in the eEF1A1b deficient and the control testis at 180 dah.

Serum 11-KT level was measured using the EIA Assay kits (Cayman Chemical Co, USA) following the manufacturers’ instructions. Blood samples were collected from the caudal vein of the eEF1A1b deficient and control XY fish at 90 dah and kept at 4 °C overnight. Serum was collected after centrifugation and stored at −20 °C until use.

Sperm collected from eEF1A1b deficient and the control XY fish at 180 dah were diluted 1:1000 with phosphate buffered saline. Examination of morphology and motility of sperm was performed under Olympus BX51 light microscope.

To test fertility, 1200 mature eggs from an XX mother fish were divided into six groups (each with 200 eggs), and artificial insemination was performed using sperm from eEF1A1b deficient and control XY fish (n = 3). 12 hours later, the number of gastrula-stage embryos was scored under a light microscope to calculate fertilization rate. Hatching rates were calculated at 1 dah (5 days post fertilization). The concentration of sperm from eEF1A1b deficient and control XY fish was calculated using cell count data from flow cytometry (n = 3).

### Production of F1

The eEF1A1b heterozygous F_1_ offspring were obtained by mating the F_0_ female founder with a wild-type male. F_1_ fish were screened by restriction enzyme digestion of PCR amplifications, as described above. Mutate sequences were confirmed by Sanger sequencing.

### Heterozygous mutant of eEF1A1b on spermatogenesis and fertility

The gonads of heterozygous mutant of *eEF1A1b (eEF1A1b*^+/−^) XY fish were sampled at 90, 120, 150 and 180 dah for H.E and IHC analysis. eEF1A1b, Oct 4 (spermatogonia maker) and Cyp11b2 antibodies were prepared by our laboratory. Phospho-histone h3 (spermatocytes maker) antibody was obtained from the Cell signaling Technology (Beverly, MA, USA). Antibodies against eEF1A1b, Oct4, Histone 3 and Cyp11b2 were diluted 1:1000, 1:1000, 1:1000 and 1:500 for use, respectively. Sf-1 and StarI antibodies were prepared by our laboratory. The specificity of Sf-1 and StarI antibodies was checked previously^48^,^53^. Antibodies against Sf-1 and StarI were diluted 1:1000 and 1:200 for use, respectively. Serum 11-KT level of *eEF1A1b*^+/−^ and *eEF1A1b*^+/+^ fish at 90 and 180 dah were measured using the EIA Assay kits as described above.

Sperm of *eEF1A1b*^+/−^ and *eEF1A1b*^+/+^ XY fish was sampled 180 dah to assay sperm quality. Fertility tests were performed as described above. Sperm analysis, including sperm count, morphological abnormality, trajectory, flagellum beat frequency, motility and path velocity, were conducted using Sperm Quality Analyzer (ZKPACS-E) in The Ninth People’s Hospital of Chongqing.

### Transcriptome analysis

The transcriptomes of 180 dah control testes was sequenced previously by our group^54^. Six testes from *eEF1A1b*^+/−^ XY fish were used for transcriptome analyses. Total RNA was extracted from testes using the RNeasy Mini Kit (50) (Qiagen) according to the manufacturer’s instructions. The extracted RNA was further treated with deoxyribonuclease 1 (ribonuclease free) to eliminate genomic DNA contamination. The oligo (dT) bead-enriched mRNA was disrupted into short fragments (200–700 nt) using fragmentation buffer. These short fragments were used as templates for first- and second-strand cDNA synthesis using a DNA synthesis kit (Invitrogen). A QiaQuick PCR purification kit (Qiagen) was used to purify these fragments, and the elution buffer was used for end repair and addition of the poly (A) tail. Then, these short fragments were ligated to sequencing adapters. After agarose gel electrophoresis, fragments between 320 and 370 nt were cut from the gel for PCR amplification. cDNA libraries were constructed from the two samples and sequenced on an Illumina HiSeq2000 instrument. Clean reads from each library were aligned to the reference genome (Orenil1.0, http://www.ensembl.org/Oreochromis_niloticus/Info/Index) using Tophat with default parameters, and the reads per kb per million reads (RPKM) method was used to calculated gene expression level^55^. The assembled transcripts were merged with the reference annotation (*Oreochromis niloticus*. Orenil1.0.78.gtf, downloaded from Ensembl) using Cuffmerge, and differential expression analysis was performed using Cuffdiff. Transcriptomes of control-XY and *eEF1A1b*^+/−^ XY were used to analyze the genes expression profiles. The threshold for the p-value was determined using false discovery rate (FDR) set at 0.01^31^. In this study, genes with RPKM < 1 in the transcriptome were considered to be background expression and were excluded from further analysis. Gonadal expressed genes were divided into non-differentially expressed genes between control and *eEF1A1b*^+/−^ XY (NDGs), *eEF1A1b*^+/−^ XY up-regulated genes (URGs), and *eEF1A1b*^+/−^ XY down-regulated genes (DRGs). ‘FDR > 10^−2^’ and ‘−1 < log2 |(control-XY_RPKM/*eEF1A1b*^+/−^ XY_RPKM)| < 1’ were used to identify NDGs, ‘FDR > 10^−2^’ and log2 (control-XY_RPKM/*eEF1A1b*^+/−^ XY_RPKM) > 1 were used to identify DRGs, and ‘FDR > 10^−2^’ and (control-XY_RPKM/*eEF1A1b*^+/−^ XY_RPKM) < −1 were used to identify (URGs). To study the biological pathways of the up/down-regulated genes involved, we mapped these differentially expressed genes to pathways in the Kyoto Encyclopedia of Genes and Genomes (KEGG) using KOBAS web server (http://kobas.cbi.pku.edu.cn/).

### Transgene mediated rescue of spermatogenesis in eEF1A1b^+/−^ XY fish

*In vivo* transgenic overexpression of the *eEF1A1b* in *eEF1A1b*^+/−^ XY individuals was performed according to the methods of our previous study^49^. An expression plasmid for *eEF1A1b* was prepared as follows. Complementary DNA encoding the ORF of *eEF1A1b* was amplified by PCR with a primer set introducing the *Bam*HI and *Eco*RI sites (Supplemental Tab. 2). The amplified fragment was digested by *Bam*HI and *Eco*RI and ligated into the multiple cloning sites downstream of the cytomegalovirus (CMV) sequence of the pIRES-hrGFP-1a vector (Stratagene, La Jolla, CA). The prepared construct was microinjected into *eEF1A1b*^+/−^ XY fertilized eggs at the one-cell stage. Genomic DNA was extracted from injected fish and used to screen transgenic fish at 90 dah. DNA fragments were amplified using gene specific primers (Supplemental Tab. 2). The testis of the injected fish was examined by monitoring the GFP signal, and later the testis was subjected to both histological and immunohistochemical analyses. Antibodies against Ph3, Vasa and Cyp11b2 were diluted as described above. Serum 11-KT level was measured using the EIA Assay kits as described above.

## Additional Information

**How to cite this article:** Chen, J. *et al*. Heterozygous mutation of eEF1A1b resulted in spermatogenesis arrest and infertility in male tilapia, *Oreochromis niloticus. Sci. Rep.* **7**, 43733; doi: 10.1038/srep43733 (2017).

**Publisher's note:** Springer Nature remains neutral with regard to jurisdictional claims in published maps and institutional affiliations.

## Supplementary Material

Supplementary Information

## References

[b1] Mateyak M. K. & Kinzy T. G. eEF1A: thinking outside the ribosome. Journal of Biological Chemistry 285, 21209–21213 (2010). 20444696 10.1074/jbc.R110.113795 PMC2898402

[b2] Koji N., Masataka K., Shigekazu N. & Yoshito K. Structure of the two genes coding for polypeptide chain elongation factor lα (EF-lα) froni Saccharomyces cerevisiae. Gene 45, 265–273 (1986). 3026912 10.1016/0378-1119(86)90024-7

[b3] Hovemann B., Richter S., Walldorf U. & Cziepluch C. Two genes encode related cytoplasmic elongation factors 1α (EF-1α) in Drosophila melanogaster with continuous and stage specific expression. Nucleic Acids Research 16, 3175–3194 (1988). 3131735 10.1093/nar/16.8.3175 PMC336487

[b4] Danforth B. N. & Ji S. Elongation factor-1 alpha occurs as two copies in bees: implications for phylogenetic analysis of EF-1 alpha sequences in insects. Molecular biology and evolution 15, 225–235 (1998). 9501490 10.1093/oxfordjournals.molbev.a025920

[b5] Gao D., Li Z., Murphy T. & Sauerbier W. Structure and transcription of the gene for translation elongation factor 1 subunit alpha of zebrafish (Danio rerio). Biochimica et Biophysica Acta (BBA)-Gene Structure and Expression 1350, 1–5 (1997). 9003448 10.1016/s0167-4781(96)00179-0

[b6] Kinoshita M. . cDNA Cloning of Polypeptide Chain Elongation Factor 1. ALPHA. from Medaka Oryzias latipes. Fisheries science 65, 133–137 (1999).

[b7] Nowell M. A. . Cloning and expression of an elongation factor-1α in sea bream (Sparus aurata) larvae and adult tissue. Marine Biotechnology 2, 173–179 (2000). 10811957 10.1007/s101269900022

[b8] Mochida K. & Matsubara T. Molecular cloning of an elongation factor 1α and its mRNA localization in testis of the Nile tilapia Oreochromis niloticus. Fisheries science 68, 830–837 (2002).

[b9] Infante C., Asensio E., Cañavate J. P. & Manchado M. Molecular characterization and expression analysis of five different elongation factor 1 alpha genes in the flatfish Senegalese sole (Solea senegalensis Kaup): differential gene expression and thyroid hormones dependence during metamorphosis. BMC molecular biology 9, 1 (2008). 18234081 10.1186/1471-2199-9-19 PMC2270864

[b10] Abdallah B., Hourdry J., Krieg P. A., Denis H. & Mazabraud A. Germ cell-specific expression of a gene encoding eukaryotic translation elongation factor 1 alpha (eEF-1 alpha) and generation of eEF-1 alpha retropseudogenes in Xenopus laevis. Proceedings of the National Academy of Sciences 88, 9277–9281 (1991). 10.1073/pnas.88.20.9277 PMC52697 1924391

[b11] Deschamps S. . Two forms of elongation factor 1 alpha (EF-1 alpha O and 42Sp50), present in oocytes, but absent in somatic cells of Xenopus laevis. The Journal of cell biology 114, 1109–1111 (1991). 1894690 10.1083/jcb.114.6.1109 PMC2289126

[b12] Loeber J., Claußen M., Jahn O. & Pieler T. Interaction of 42Sp50 with the vegetal RNA localization machinery in Xenopus laevis oocytes. FEBS Journal 277, 4722–4731 (2010). 20977669 10.1111/j.1742-4658.2010.07878.x

[b13] Lee S., Francoeur A.-M., Liu S. & Wang E. Tissue-specific expression in mammalian brain, heart, and muscle of S1, a member of the elongation factor-1 alpha gene family. Journal of Biological Chemistry 267, 24064–24068 (1992). 1385435

[b14] Knudsen S. M., Frydenberg J., Clark B. F. & Leffers H. Tissue-dependent variation in the expression of elongation factor-1α isoforms: Isolation and characterisation of a cDNA encoding a novel variant of human elongation-factor 1α. European Journal of Biochemistry 215, 549–554 (1993). 8354261 10.1111/j.1432-1033.1993.tb18064.x

[b15] Lee S., Wolfraim L. A. & Wang E. Differential expression of S1 and elongation factor-1 alpha during rat development. Journal of Biological Chemistry 268, 24453–24459 (1993). 8226996

[b16] Lee S., LeBlanc A., Duttaroy A. & Wang E. Terminal differentiation-dependent alteration in the expression of translation elongation factor-1α and its sister gene, S1, in neurons. Experimental cell research 219, 589–597 (1995). 7641810 10.1006/excr.1995.1268

[b17] Chambers D. M., Peters J. & Abbott C. M. The lethal mutation of the mouse wasted (wst) is a deletion that abolishes expression of a tissue-specific isoform of translation elongation factor 1α, encoded by the Eef1a2 gene. Proceedings of the National Academy of Sciences 95, 4463–4468 (1998). 10.1073/pnas.95.8.4463 PMC22512 9539760

[b18] Khalyfa A. . Characterization of elongation factor-1A (eEF1A-1) and eEF1A-2/S1 protein expression in normal and wasted mice. Journal of Biological Chemistry 276, 22915–22922 (2001). 11294870 10.1074/jbc.M101011200

[b19] Newbery H. . Translation elongation factor eEF1A2 is essential for post-weaning survival in mice. Journal of Biological Chemistry 282, 28951–28959 (2007). 17640869 10.1074/jbc.M703962200

[b20] Gao Y. . Mammalian elongation factor 4 regulates mitochondrial translation essential for spermatogenesis. Nature structural & molecular biology 23, 441–449 (2016). 10.1038/nsmb.3206 27065197

[b21] Shiina N., Gotoh Y., Kubomura N., Iwamatsu A. & Nishida E. Microtubule severing by elongation factor 1 alpha. Science 266, 282–285 (1994). 7939665 10.1126/science.7939665

[b22] Yang F., Demma M., Warren V., Dharmawardhane S. & Condeelis J. Identification of an actin-binding protein from Dictyostelium as elongation factor 1a. Nature 347, 494–496 (1990). 2215665 10.1038/347494a0

[b23] Abbas W., Kumar A. & Herbein G. The eEF1A proteins: at the crossroads of oncogenesis, apoptosis, and viral infections. Frontiers in oncology 5 (2015). 10.3389/fonc.2015.00075 PMC4387925 25905039

[b24] Hotokezaka Y. . Interaction of the eukaryotic elongation factor 1A with newly synthesized polypeptides. Journal of Biological Chemistry 277, 18545–18551 (2002). 11893745 10.1074/jbc.M201022200

[b25] Chuang S.-M. . Proteasome-mediated degradation of cotranslationally damaged proteins involves translation elongation factor 1A. Molecular and cellular biology 25, 403–413 (2005). 15601860 10.1128/MCB.25.1.403-413.2005 PMC538794

[b26] Tash J. S. . Gamendazole, an orally active indazole carboxylic acid male contraceptive agent, targets HSP90AB1 (HSP90BETA) and EEF1A1 (eEF1A), and stimulates Il1a transcription in rat Sertoli cells. Biology of reproduction 78, 1139–1152 (2008). 18218611 10.1095/biolreprod.107.062679

[b27] Li M. . Efficient and heritable gene targeting in tilapia by CRISPR/Cas9. Genetics 197, 591–599 (2014). 24709635 10.1534/genetics.114.163667 PMC4063917

[b28] Wosnitzer M. S. . Ubiquitin specific protease 26 (USP26) expression analysis in human testicular and extragonadal tissues indicates diverse action of USP26 in cell differentiation and tumorigenesis. PloS one 9, e98638 (2014). 24922532 10.1371/journal.pone.0098638 PMC4055479

[b29] Wargelius A. . Dnd knockout ablates germ cells and demonstrates germ cell independent sex differentiation in Atlantic salmon. Scientific reports 6 (2016). 10.1038/srep21284 PMC4758030 26888627

[b30] Wang Y. . Prmt5 is required for germ cell survival during spermatogenesis in mice. Scientific reports 5 (2015). 10.1038/srep11031 PMC4466585 26072710

[b31] Tao W. . Characterization of gonadal transcriptomes from Nile tilapia (Oreochromis niloticus) reveals differentially expressed genes. PloS one 8, e63604 (2013). 23658843 10.1371/journal.pone.0063604 PMC3643912

[b32] Sun Y.-L. . Screening and characterization of sex-linked DNA markers and marker-assisted selection in the Nile tilapia (Oreochromis niloticus). Aquaculture 433, 19–27 (2014).

[b33] Brawand D. . The genomic substrate for adaptive radiation in African cichlid fish. Nature 513, 375–381 (2014). 25186727 10.1038/nature13726 PMC4353498

[b34] Taylor J. S., Braasch I., Frickey T., Meyer A. & Van de Peer Y. Genome duplication, a trait shared by 22,000 species of ray-finned fish. Genome research 13, 382–390 (2003). 12618368 10.1101/gr.640303 PMC430266

[b35] Munshi R. . Overexpression of translation elongation factor 1A affects the organization and function of the actin cytoskeleton in yeast. Genetics 157, 1425–1436 (2001). 11290701 10.1093/genetics/157.4.1425 PMC1461591

[b36] Gross S. R. & Kinzy T. G. Translation elongation factor 1A is essential for regulation of the actin cytoskeleton and cell morphology. Nature structural & molecular biology 12, 772–778 (2005). 10.1038/nsmb979 16116436

[b37] Mu W., Starmer J., Fedoriw A. M., Yee D. & Magnuson T. Repression of the soma-specific transcriptome by Polycomb-repressive complex 2 promotes male germ cell development. Genes & development 28, 2056–2069 (2014). 25228648 10.1101/gad.246124.114 PMC4173155

[b38] Zheng B. . Establishment of a proteomic profile associated with gonocyte and spermatogonial stem cell maturation and differentiation in neonatal mice. Proteomics 14, 274–285 (2014). 24339256 10.1002/pmic.201300395

[b39] Pulvers J. N. . Mutations in mouse Aspm (abnormal spindle-like microcephaly associated) cause not only microcephaly but also major defects in the germline. Proceedings of the National Academy of Sciences 107, 16595–16600 (2010). 10.1073/pnas.1010494107 PMC2944708 20823249

[b40] Miura C., Miura T., Yamashita M., Yamauchi K. & Nagahama Y. Hormonal induction of all stages of spermatogenesis in germ-somatic cell coculture from immature Japanese eel testis. Development, growth & differentiation 38, 257–262 (1996). 10.1046/j.1440-169X.1996.t01-2-00004.x 37280997

[b41] M Escott G., A da Rosa L. & da Silveira Loss E. Mechanisms of Hormonal Regulation of Sertoli Cell Development and Proliferation: A Key Process for Spermatogenesis. Current molecular pharmacology 7, 96–108 (2014). 25620228 10.2174/1874467208666150126155032

[b42] Manova K., Nocka K., Besmer P. & Bachvarova R. F. Gonadal expression of c-kit encoded at the W locus of the mouse. Development 110, 1057–1069 (1990). 1712701 10.1242/dev.110.4.1057

[b43] Rios-Rojas C., Spiller C., Bowles J. & Koopman P. Germ cells influence cord formation and leydig cell gene expression during mouse testis development. Developmental Dynamics (2015). 10.1002/dvdy.24371 26637965

[b44] Qiu Y. . Figla Favors Ovarian Differentiation by Antagonizing Spermatogenesis in a Teleosts, Nile Tilapia (Oreochromis niloticus). PloS one 10, e0123900 (2015). 25894586 10.1371/journal.pone.0123900 PMC4404364

[b45] Curi S. . Asthenozoospermia: analysis of a large population. Archives of andrology 49, 343–349 (2003). 12893510 10.1080/01485010390219656

[b46] Tamura K. . MEGA5: molecular evolutionary genetics analysis using maximum likelihood, evolutionary distance, and maximum parsimony methods. Molecular biology and evolution 28, 2731–2739 (2011). 21546353 10.1093/molbev/msr121 PMC3203626

[b47] Livak K. J. & Schmittgen T. D. Analysis of relative gene expression data using real-time quantitative PCR and the 2−ΔΔCT method. methods 25, 402–408 (2001). 11846609 10.1006/meth.2001.1262

[b48] Yu X. . Characterization of two paralogous StAR genes in a teleost, Nile tilapia (Oreochromis niloticus). Molecular and cellular endocrinology 392, 152–162 (2014). 24859646 10.1016/j.mce.2014.05.013

[b49] Wang D.-S. . Foxl2 up-regulates aromatase gene transcription in a female-specific manner by binding to the promoter as well as interacting with ad4 binding protein/steroidogenic factor 1. Molecular Endocrinology 21, 712–725 (2007). 17192407 10.1210/me.2006-0248

[b50] Ostyn A., De Buyser M. L., Guillier F., Krys S. & Hennekinne J. A. Benefits of the combined use of immunological-and PCR-based methods for determination of staphylococcal enterotoxin food safety criteria in cheeses. Food Analytical Methods 5, 173–178 (2012).

[b51] Kobayashi T., Kajiura-Kobayashi H. & Nagahama Y. Two isoforms of vasa homologs in a teleost fish: their differential expression during germ cell differentiation. Mechanisms of development 111, 167–171 (2002). 11804791 10.1016/s0925-4773(01)00613-x

[b52] Zhang X. . Isolation of doublesex-and Mab-3-related transcription factor 6 and its involvement in spermatogenesis in tilapia. Biology of reproduction, biolreprod. 114.121418 (2014). 10.1095/biolreprod.114.121418 25320148

[b53] Xie Q.-P. . Haploinsufficiency of SF-1 Causes Female to Male Sex Reversal in Nile Tilapia, Oreochromis niloticus. Endocrinology, en. 2015–2049 (2016). 10.1210/en.2015-2049 27046435

[b54] Sun L.-N. . Transdifferentiation of differentiated ovary into functional testis by long-term treatment of aromatase inhibitor in Nile tilapia. Endocrinology 155, 1476–1488 (2014). 24437491 10.1210/en.2013-1959

[b55] Mortazavi A., Williams B. A., McCue K., Schaeffer L. & Wold B. Mapping and quantifying mammalian transcriptomes by RNA-Seq. Nature methods 5, 621–628 (2008). 18516045 10.1038/nmeth.1226 PMC13303166

