# Supplementary material for: Heterozygous mutation of eEF1A1b resulted in spermatogenesis arrest and infertility in male tilapia, Oreochromis niloticus
Source: Sci Rep. 2017 Mar 7;7:43733. doi: 10.1038/srep43733 (PMC5339811; doi:10.1038/srep43733)

**Heterozygous mutation of eEF1A1b resulted in spermatogenesis arrest and infertility in male tilapia, *Oreochromis niloticus***

## Jinlin Chen, Dongneng Jiang, Dejie Tan, Zheng Fan, Yingying Wei, Minghui Li, Deshou Wang*

**Supplemental Tables**

**Supplemental Table. S1. The accession numbers of eEF1A1 sequences used in phylogenetic analysis**

| Gene | Accession Number | Gene | Accession Number |
| --- | --- | --- | --- |
| Human-eEF1A1 | NP_001393.1 | Zebrafish-eEF1A1a | NP_001017795.1 |
| Rat--eEF1A1 | NP_034236.2 | Zebrafish-eEF1A1b | NP_571338.1 |
| Chicken-eEF1A1 | NP_989488.2 | Medaka-eEF1A1a | XP_004077036.1 |
| green anole -eEF1A1 | XP_003226101.1 | Medaka-eEF1A1b | XP_004074126.1 |
| clawed frog -eEF1A1 | NP_001016692.1 | Fugu-eEF1A1a | XP_003963863.1 |
| Tilapia-eEF1A1a | XP_003438367.1 | Fugu-eEF1A1b | NP_001032962.1 |
| Tilapia-eEF1A1b | XP_005470211.1 | Shark-eEF1A1b | NP_001279518.1| |
| Tetraodon-eEF1A1b | ENSTNIP00000012710 | Tilapia-42Sp50 | XP_003439294.1| |
| Spotted-gar-eEF1A1b | XP_006638682.1| |  |  |

**Supplemental Table. S2. Sequences of primers used in the present study**

| Primer | Sequence | Amplification efficiency | Purpose |
| --- | --- | --- | --- |
| *eEF1A1b*-gRNA-F | TAATACGACTCACTATAGGGATTATCGTCCCGAAGAAGTTTTAGAGCTAGAAATAGC |  | *eEF1A1b* gRNA amplification |
| gRNA-R | AGCACCGACTCGGTGCCAC |  |  |
| *eEF1A1b*-cas-F1 | GTTGTTCCCTTTATGAAGATGATCC |  | Fragment amplification |
| *eEF1A1b*-cas-R1 | CAATGATGGTGACGTAATACTTGCT |  |  |
| *eEF1A1b*-TG-F | CGCGGATCCATGGGGAAGGAGAAGCTCCACATC |  |  |
| *eEF1A1b*-TG-R | CCGGAATTCTTTGGCCTTCTGCGCCTTCTG |  |  |
| GFP-F | ATGGTGAGCAAGCAGATCCTG |  |  |
| GFP-R | TTACACCCACTCGTGCAGGCTGC |  |  |
| *eEF1A1b*-RT-R1 | CCGCAGAGAACTGTAACGCAAACC |  | RT-PCR |
| *eEF1A1b*-RT-R2 | GTGAATCACTAGCCGACGGCAGC |  |  |
| *eEF1A1b*-Q-R1 | CCGCAGAGAACTGTAACGCAAACC | 99.862% | Real-time PCR |
| *eEF1A1b*-Q-R2 | GTGAATCACTAGCCGACGGCAGC |  |  |
| *cyp11b2*-Q-F | CAAAGAAGTCCTCAGGTTGTACCCA | 100.958% |  |
| *cyp11b2*-Q-R | GGACCAAAGTTCCAGCAGGTATGT |  |  |
| *Sf-1*-Q-F | CATACGACGAGGACCTGGAA | 100.425% |  |
| *Sf-1*-Q-R | GAGGCATTTCTGGAACCGAC |  |  |
| *suz12a*-Q-F | AGATGGAAGTGGACAGCGAGGA | 98.576% |  |
| *suz12a* -Q-R | GGCAGCGTGGTGTTCAGCAA |  |  |
| *starI*-Q-F | CTGAAACTGTTGCTGCGAATGGA | 98.752% |  |
| *starI* -Q-R | GGTCTCTGCGGATACCTCGTG |  |  |
| *cyp11a1*-Q-F | GAAACACTCAGGTTGCATCCG | 98.587% |  |
| *cyp11a1* -Q-R | CATACAGCCCTAATTGGACCAGAG |  |  |
| *usp26*-Q-F | GCCGAATATTACGCTGGGATGA | 100.186% |  |
| *usp26*-Q-R | AACCTTACCTCCGCTGGTGAG |  |  |
| *spo11-Q-F* | ACTGGCAGCGTGCTGAAA | 99.596% |  |
| *spo11-Q-R* | CACTGTCAAAGCTTACGTTGGC |  |  |
| *β-actin*-Q-F | GGCATCACACCTTCTACAACGA | 100.097% |  |
| *β-actin-*Q-R | ACGCTCTGTCAGGATCTTCA |  | Internal control |
| *gapdh*-Q-F | AAGCTCATTTCCTGGTAT | 97.239% |  |
| *gapdh*-Q-R | CCTTTGCTGATTTCCTTG |  |  |
| *eEF1A1a-*Q-F | CAAGTGCGGAGGAATCGA | 97.856% |  |
| *eEF1A1a-*Q-R | CGAACTTCCACAGAGCGATA |  |  |

**Supplemental Table. S3. Mutation rates of eEF1A1b in each mutant XY F_0_ fish induced by CRISPR/Cas9**

| **Number**  **of fish**  **analyzed** | **Number**  **of**  **Mutants** | **Freq**  **uenc**  **y** | **Indel mutation frequency (%）** | | | | | | | | | | | | | | | | | | |
| --- | --- | --- | --- | --- | --- | --- | --- | --- | --- | --- | --- | --- | --- | --- | --- | --- | --- | --- | --- | --- | --- |
|  |  |  | **#1** | **#2** | **#3** | **#4** | **#5** | **#6** | **#7** | **#8** | **#9** | **#10** | **#11** | **#12** | **#13** | **#14** | **#15** | **#16** | **#17** | **#18** | **#19** |
| 20 | 19 | 95 | 91 | 97 | 93 | 93 | 97 | 95 | 90 | 91 | 93 | 93 | 97 | 90 | 91 | 91 | 94 | 90 | 93 | 97 | 97 |

| **Number**  **of Go**  **analyzed** | **Number**  **of**  **Mutants** | **Freq**  **uenc**  **y** | **Indel mutation frequency (%)** | | | | | | | | | | | | | | | | | | |
| --- | --- | --- | --- | --- | --- | --- | --- | --- | --- | --- | --- | --- | --- | --- | --- | --- | --- | --- | --- | --- | --- |
|  |  |  | **#20** | **#21** | **#22** | **#23** | **#24** | **#25** | **#26** | **#27** | **#28** | **#29** | **#30** | **#31** | **#32** | **#33** | **#34** | **#35** | **#36** | **#37** | **#38** |
| 20 | 19 | 95 | 90 | 93 | 96 | 93 | 93 | 91 | 96 | 95 | 93 | 95 | 90 | 92 | 90 | 91 | 94 | 93 | 92 | 91 | 94 |

**Supplemental Table. S4 Fertilization and survive rate of the control and F_0_ , F_1_ eEF1A1b mutant XY fish**

|  | **Number of two cell embryos** | **Number of**  **unfertilized oocytes** | **Fertilization rate(%)** | **Survive** | **Survive rate (%)** |
| --- | --- | --- | --- | --- | --- |
| **Control XY** | 195 | 5 | 97.5 | 155 | 77.5 |
|  | 190 | 10 | 95 | 170 | 85 |
|  | 200 | 0 | 100 | 180 | 90 |
| **F0 XY** | 45 | 135 | 22.5 | 1 | 0.5 |
|  | 25 | 175 | 12.5 | 0 | 0 |
|  | 11 | 189 | 5.5 | 0 | 0 |
| **F1 XY** | 27 | 173 | 13.5 | 5 | 2.5 |
|  | 29 | 171 | 14.5 | 7 | 3.5 |
|  | 16 | 184 | 8.0 | 2 | 1 |

**Supplemental Table. S5 *eEF1A1b*^+/-^ XY displayed spermatogenesis arrest and infertile**

| dah | WT-XY | Phenotype | H-XY | | Phenotype | |
| --- | --- | --- | --- | --- | --- | --- |
|  |  |  | -4 bp | -13bp | -4 bp | -13bp |
| 90 | 6 | NS | 11 | 8 | SA | SA |
| 120 | 3 | NS | 8 | 9 | SA | SA |
| 150 | 3 | NS | 6 | 6 | SA | SA |
| 180 | 8 | fertile | 12 | 10 | infertile | infertile |

dah, day after hatching; WT, wild type; H, heterozygous mutant; NS, normal spermatogenesis; SA, spermatogenesis arrest.

**Supplemental Figure legends**

**Supplemental FIG. S1.** **Phylogenetic analyses of eEF1A1 in vertebrates.** Phylogenetic tree of eEF1A1 proteins from human, rat, chicken, green anole, clawed frog, zebrafish, fugu, medaka and tilapia was constructed using tilapia 42Sp50 as outgroup. The values on the tree represent bootstrap scores out of 1000 trials, indicating the credibility of each branch. Branch lengths are proportional to the number of amino acid changes.

**Supplemental FIG. S2. Synteny analysis of eEF1A1 in vertebrates.** *eEF1A1* and its adjacent genes in human, rat, chicken, green anole, clawed frog, tetraodon, zebrafish, fugu and tilapia were analyzed. Rectangles represent genes in chromosome/scaffold, dotted lines represent omitted genes of the chromosome/scaffold, and the arrow represents the orientation of the gene.

**Supplemental FIG. S3.** **Expression levels of eEF1A1b in tissues of tilapia by immunohistochemistry.** eEF1A1b was expressed in the brain, heart, liver, intestine, kidney, testis and ovary. A-K, brain; gill; heart, spleen, head kidney, liver, intestine, kidney, muscle, testis and ovary, respectively. Brown color, positive signals.

**Supplemental FIG. S4.** **Sanger sequencing results of the 20 mutant XY fish and the frequency in mutation types.** Insertions are marked in green, deletions are marked by dashes, and the PAM is marked in light orange. Numbers to the right of the sequences indicate the loss or gain of bases for each allele, with the number of bases inserted (+) or deleted (−). The frequency of each mutation types were indicated in parentheses. WT, wild type.

**Supplemental FIG. S5. Measurement of sperms flagella length of *eEF1A1b* deficient F_0_ and control XY fish.** Results were expressed as mean ± SD. Different letters indicate statistical differences at P<0.05 as determined by one-way ANOVA followed by post hoc test.

**Supplemental FIG. S6.** **Histological observations of testis from XY *eEF1A1b* deficient and control fish at 120 and 180 dah.** E-F and G-H, High magnification of the boxed areas in A-B and C-D, respectively. OG, oogonia; OC, oocyte; I, phase one oocyte; II, phase II oocyte; III, phase III oocyte.

**Supplemental FIG. S7.** **Morphology of tissues with the high eEF1A1b abundance from** ***eEF1A1b*^+/-^ XY fish.** A and B, macrograph of whole body; C-F, macrograph and hematoxylin and eosin of the heart; G-J, macrograph and hematoxylin and eosin of the brain in *eEF1A1b*^+/+^ and *eEF1A1b***^+/-^** XY fish.

**Supplemental FIG. S1.**

**
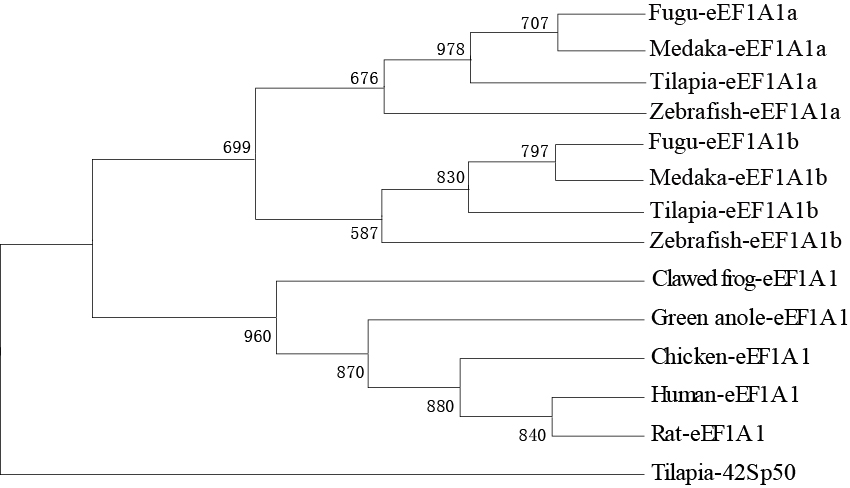
**

**Supplemental FIG. S2.**


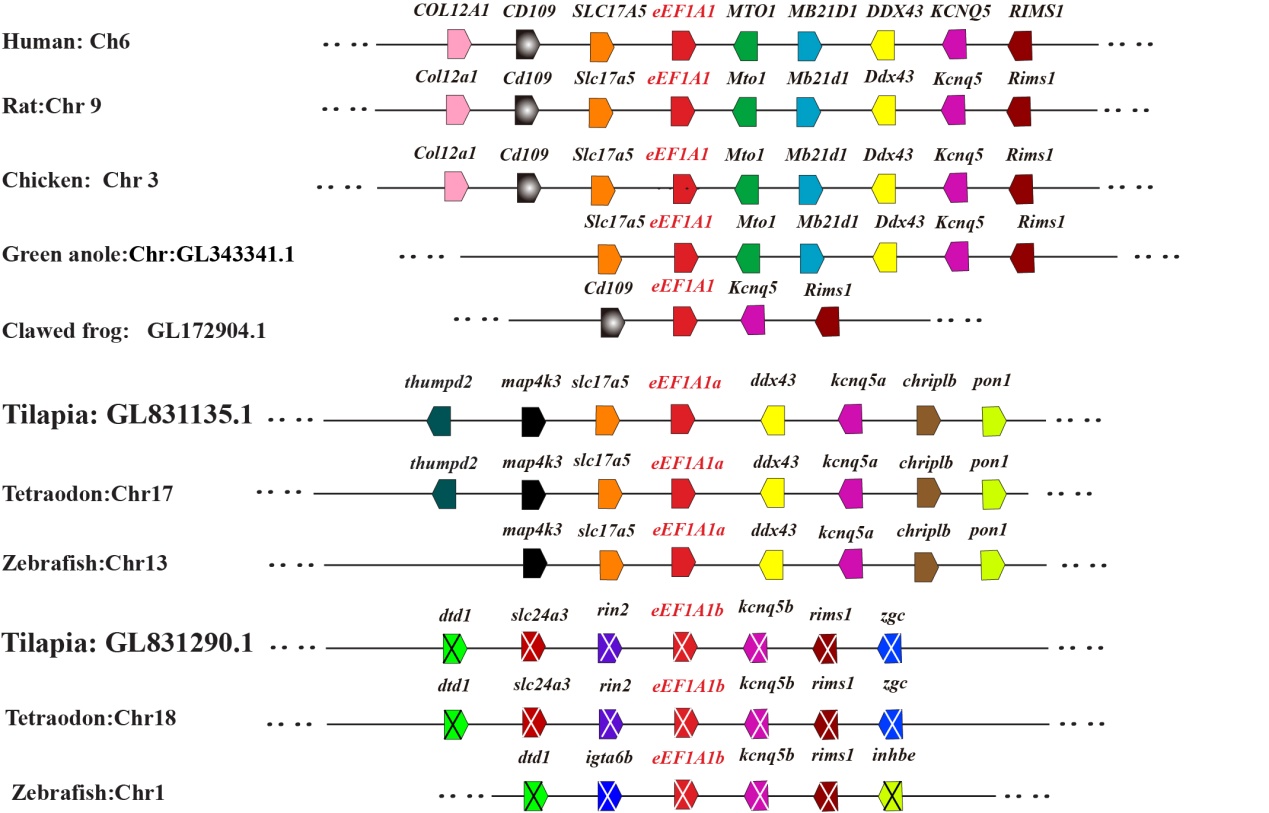


**Supplemental FIG. S3.**


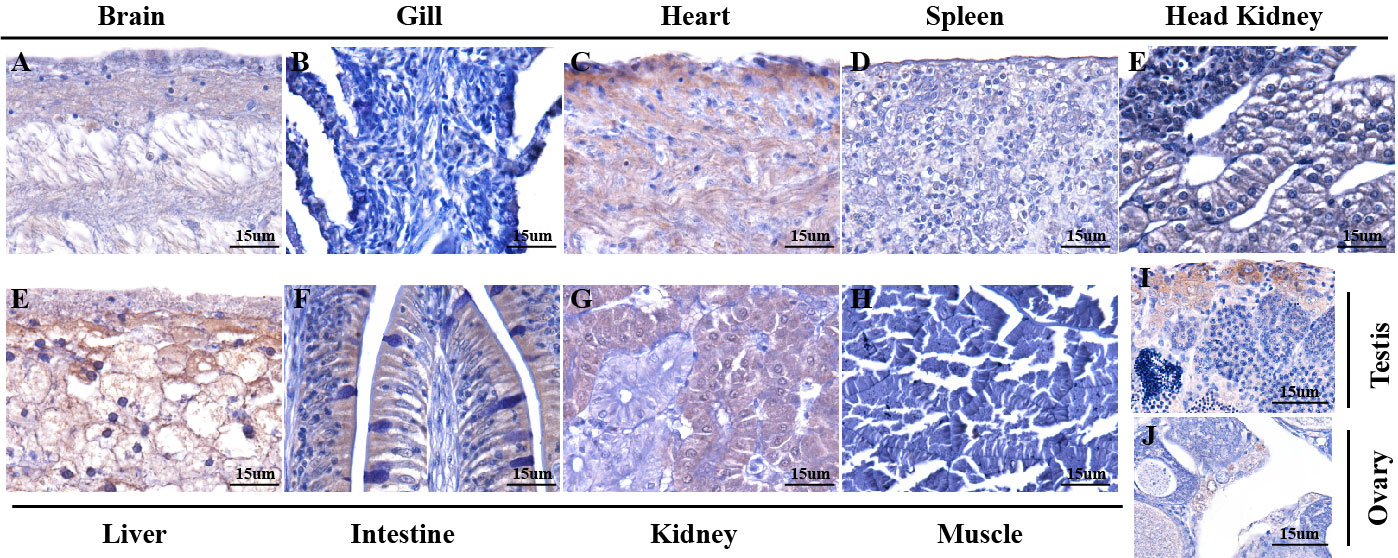


**Supplemental FIG. S4.**

**
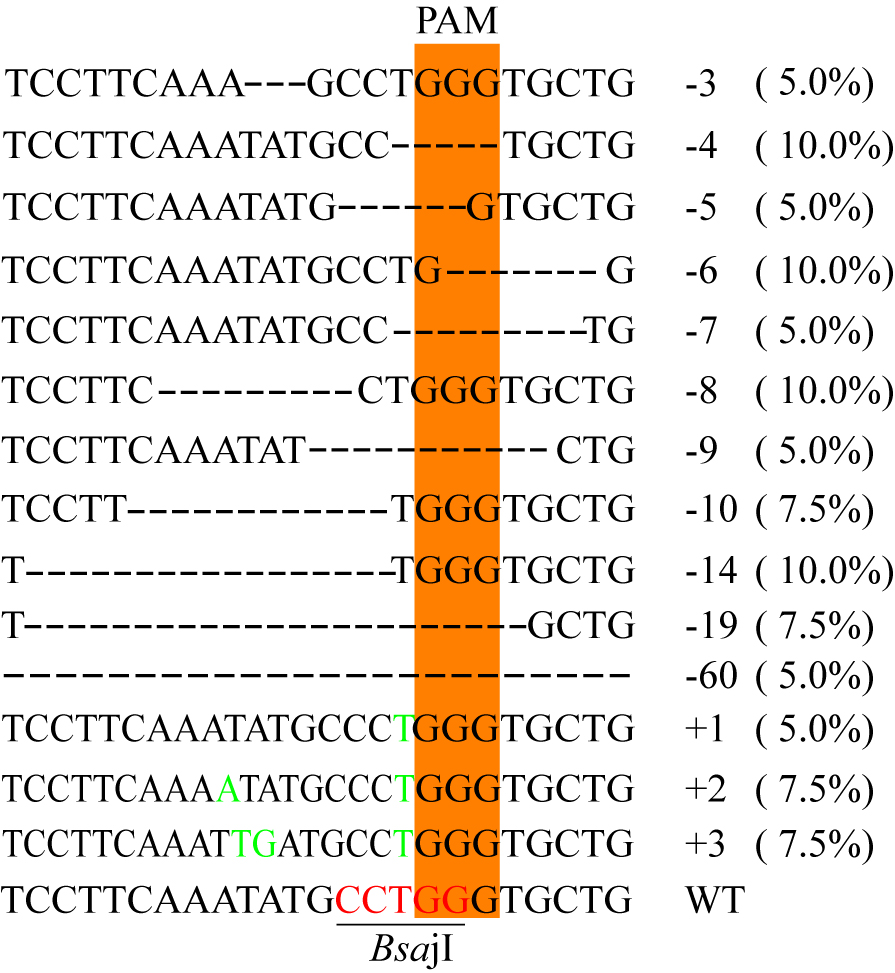
**

**Supplemental FIG. S5.**

**
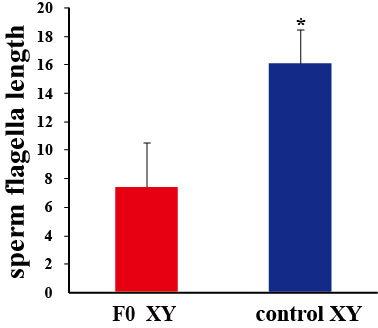
**

**Supplemental FIG. S6.**


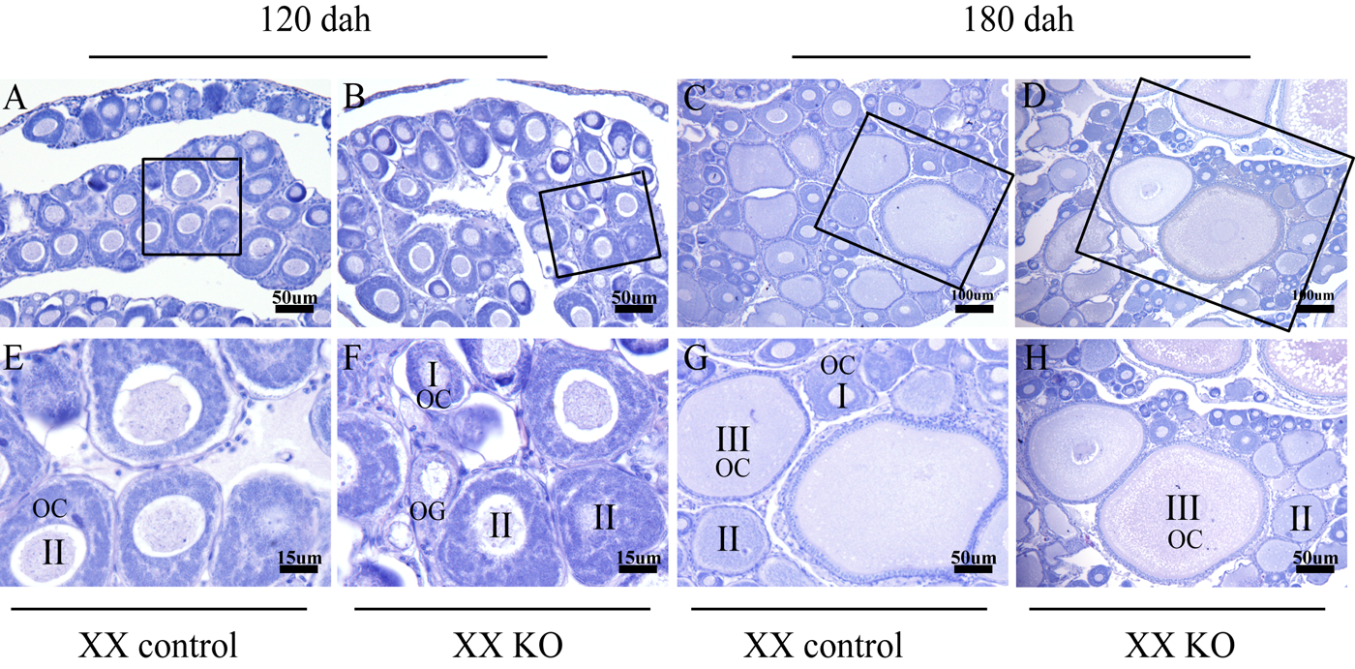


**Supplemental FIG. S7.**


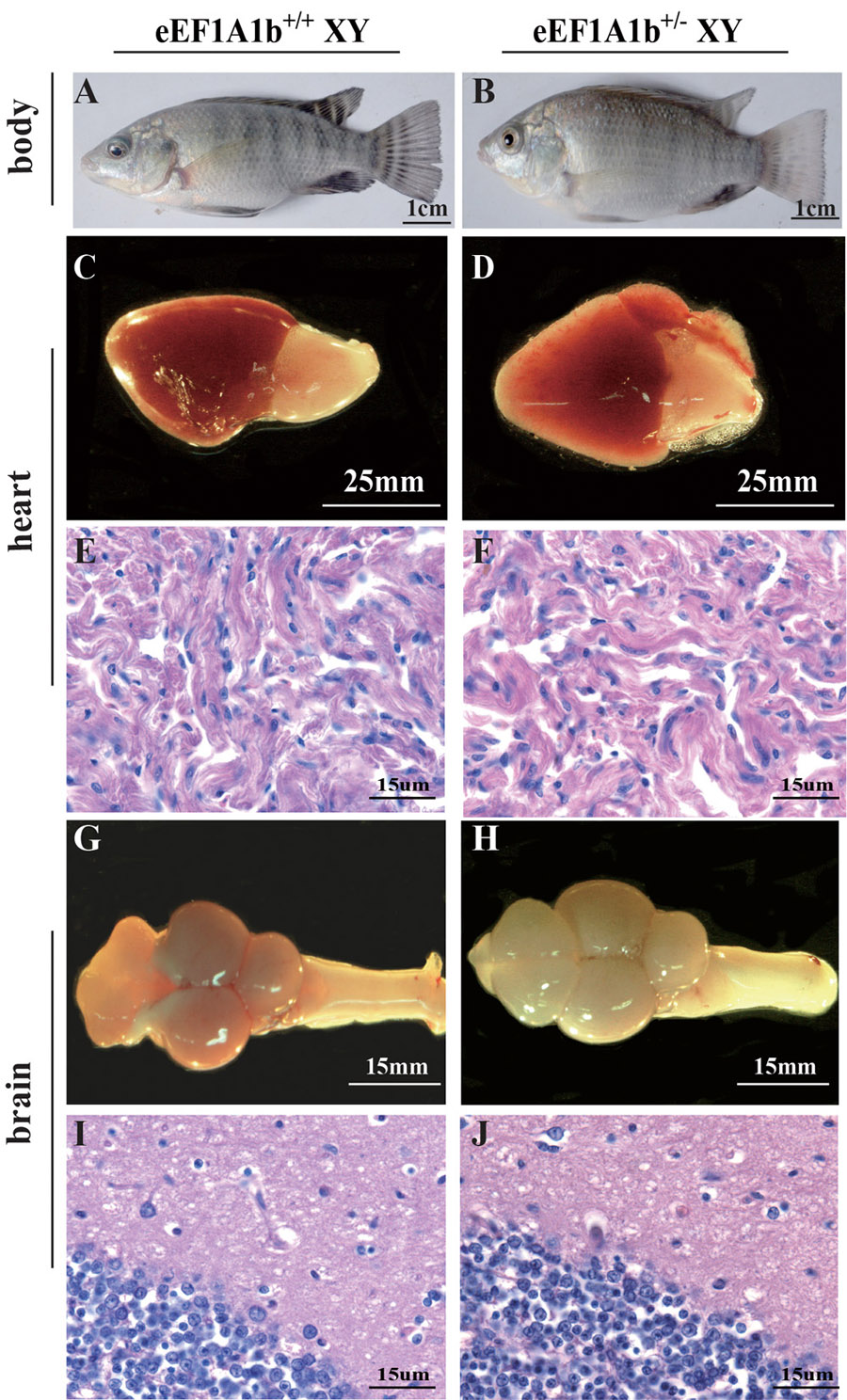

Supplement: Supplementary Information [file srep43733-s1.doc]
